# Transcriptomic profiling of microglia and astrocytes throughout aging

**DOI:** 10.1186/s12974-020-01774-9

**Published:** 2020-04-01

**Authors:** Jie Pan, Nana Ma, Bo Yu, Wei Zhang, Jun Wan

**Affiliations:** 1 Shenzhen Key Laboratory for Neuronal Structural Biology, Biomedical Research Institute, Shenzhen Peking University - The Hong Kong University of Science and Technology Medical Center, Shenzhen, Guangdong Province China; 2 Shenzhen Key Laboratory for Translational Medicine of Dermatology, Biomedical Research Institute, Shenzhen Peking University - The Hong Kong University of Science and Technology Medical Center, Shenzhen, Guangdong Province China; 3 Department of Dermatology, Peking University Shenzhen Hospital, Shenzhen, Guangdong Province China; 4 Division of Life Science, The Hong Kong University of Science and Technology, Clear Water Bay Road, Kowloon, Hong Kong, China

**Keywords:** Microglia, Astrocyte, Alzheimer’s disease (AD), Aging, RNA-seq

## Abstract

**Background:**

Activation of microglia and astrocytes, a prominent hallmark of both aging and Alzheimer’s disease (AD), has been suggested to contribute to aging and AD progression, but the underlying cellular and molecular mechanisms are largely unknown.

**Methods:**

We performed RNA-seq analyses on microglia and astrocytes freshly isolated from wild-type and APP-PS1 (AD) mouse brains at five time points to elucidate their age-related gene-expression profiles.

**Results:**

Our results showed that from 4 months onward, a set of age-related genes in microglia and astrocytes exhibited consistent upregulation or downregulation (termed “age-up”/“age-down” genes) relative to their expression at the young-adult stage (2 months). And most age-up genes were more highly expressed in AD mice at the same time points. Bioinformatic analyses revealed that the age-up genes in microglia were associated with the inflammatory response, whereas these genes in astrocytes included widely recognized AD risk genes, genes associated with synaptic transmission or elimination, and peptidase-inhibitor genes.

**Conclusions:**

Overall, our RNA-seq data provide a valuable resource for future investigations into the roles of microglia and astrocytes in aging- and amyloid-β-induced AD pathologies.

## Background

Aging and Alzheimer’s disease (AD) produce widespread effects on the central nervous system (CNS) that are characterized by cognitive decline, vulnerability to physical illnesses, elevated oxidative stress, and chronic brain inflammation [1]. These biological and pathological processes are also associated with diminished blood-brain barrier (BBB) integrity, which leads to the accumulation in the brain of blood-derived proteins [2, 3] and the infiltration of peripheral cells [4–9], and multiple lines of evidence indicate that the innate-immune functions of microglia and astrocytes are involved in these processes.

Microglia, the resident macrophages in the CNS, are originally derived from primitive myeloid progenitors that are seeded in the brain during fetal development and expand drastically after birth to account for 5–12% of all the cells in the brain [10–13]. In the CNS, microglia play crucial roles in the maintenance of brain homeostasis by regulating synaptic plasticity, remodeling neuronal circuits, defending against infectious pathogens [13–15], and promoting tip-cell fusion to participate in angiogenesis [16]. In aging and in mice with AD, microglial activation in the brain acts a double-edged sword: Although activated microglia facilitate the phagocytosis and clearance of infectious agents or amyloid-β (Aβ) deposits, constant exposure to proinflammatory cytokines exerts detrimental effects on the brain [17, 18]. As compared to microglia, astrocytes, which constitute another type of glial cells in the CNS, have been less studied in aging and AD pathogenesis. Historically, astrocytes have been considered as supportive cells that either provide nitrites or serve as physical scaffolds for neurons. The perivascular endfeet of astrocytes ensheath 98% of brain parenchymal capillaries and thus contribute to BBB integrity and maintain osmotic homeostasis and gliovascular signaling [19, 20]. Astrocytes have to date been documented to play essential roles in neurophysiology, such as in release of gliotransmitters (glucose, ATP, and glutamate), communication with neurons, and modulation of synaptic structure [21].

Over the few past decades, considerable research effort has been devoted toward elucidating the functions of microglia and astrocytes in the brain under both physiological and pathological conditions. Moreover, in previous studies, RNA-sequencing (RNA-seq) analysis of microglia and astrocytes has been performed in geriatric and young mice to identify the transcriptomic alterations that occur during aging [22, 23]; however, in these studies, the sequencing samples were collected either at limited time points or over large time intervals, and thus the genes that were identified to be altered in aged mice could have been affected by unknown/unexpected insults that are not related to aging or specific diseases. Therefore, to clarify the effects of factors associated with late-age disorders of the CNS, we investigated aging-related genes in microglia and astrocytes isolated from the mouse brain at 5 time points. Here, we identify 2 age-related gene clusters whose expression increased with age as compared with the expression in mature-adult mice. Differential gene-expression analysis revealed that inflammatory-response genes constituted the most prominent class of consistently upregulated genes in microglia upon aging, whereas in astrocytes, synaptic-transmission/elimination- and peptidase-inhibitor-related genes were most markedly increased. Furthermore, most of the aging-related genes also showed notable differences in AD mice relative to their expression in wild-type (WT) mice. Our results thus provide a novel transcriptomic dataset for microglia and astrocytes throughout aging that could offer new insights into the body’s early intrinsic mechanisms involved in sensing CNS damage and protecting the brain against neurodegeneration.

## Material and methods

### Mice

APPswe/PS1ΔE9 double-transgenic mice, obtained from the Model Animal Research Center of Nanjing University (Nanjing, China), were originated from B6.Cg-Tg (APPswe/PS1ΔE9) 85Dbo/Mmjax mice (JAX#034832) of the Jackson Laboratory. C57BL/6 J WT littermates were used as WT controls. Mice (*n* = 3/group) were bred under SPF conditions in IVC cages at 23 °C and 50–60% humidity and with circadian-rhythm illumination. Pups aged 21–28 days old were removed from their parental cages and genotyped using ear-biopsy samples; the DNA extracted from the biopsy samples was PCR-amplified using primers specific for APP and PS1 sequences. All procedures were approved by the Animal Use and Care Committee of Shenzhen Peking University - The Hong Kong University of Science and Technology Medical Center (SPHMC) (protocol number 2011-004). All mice used in the study were males. Efforts were made to minimize suffering and the number of animals used.

### Brain dissociation

Microglia and astrocytes were isolated from WT and AD mice belonging to 5 age groups: 2-, 4-, 6-, 9-, and 12-month old (2–12 months). Mice were transcardially perfused under deep anesthesia with 1 × PBS, and then the brain was removed, dissected, and rinsed in HBSS. Next, after removing the meninges, the brain was cut into small pieces by using a sterile scalpel, and the samples were centrifuged at 300×*g* for 2 min at room temperature and the supernatant was aspirated carefully. Samples from a single brain were pooled as a single experimental group. Enzymatic cell dissociation was performed using an Adult Brain Dissociation Kit (130-107-677, Miltenyi Biotec), according to the manufacturer’s instructions. Briefly, tissue pieces (up to 500 mg of tissue per sample) were transferred into the C Tube containing 1950 μL of enzyme mix 1 (enzyme P and buffer Z), and then 30 μL of enzyme mix 2 (enzyme A and buffer Y) was added into the C Tube. The C Tube was tightly closed and attached upside down onto the sleeve of the gentleMACS Octo Dissociator with Heaters (130-096-427, Miltenyi Biotec), and the appropriate gentleMACS program was run. After brief centrifugation to collect samples at the tube bottom, the samples were filtered through a 70-μm strainer (130-098-462, Miltenyi Biotec), washed with D-PBS, and then centrifuged again.

### Percoll density gradient and myelin removal

Singe cells were resuspended in 40% Percoll and centrifuged at 800×*g* for 20 min at 15 °C. After discarding the myelin-containing supernatant, the pellet was resuspended in cold MACS buffer (containing 1-volume dilution of PBS, 2 mM EDTA, and 0.5% BSA, pH 7.2), and then myelin-removal beads (Myelin Removal Beads II, 130-96-733, Miltenyi Biotec) were used according to the manufacturer’s protocol to prepare cells for staining with fluorescence activated cell sorting (FACS) antibodies. Briefly, single-cell suspensions were incubated with the beads at 4 °C for 15 min, and then the cells were washed onto the LS column on the autoMACS Separator; the column was washed thrice with PB buffer, and the cells in the flow-through were used for antibody staining.

### FACS sorting of microglia and astrocytes

After isolation, cell pellets were resuspended in FC receptor blocking solution (553141, BD Biosciences), incubated on ice for 10 min, and costained for 30 min on ice in the dark with PE-Cy7-labeled CD45 (103114, BioLegend), PE-labeled CD11b (101208, BioLegend), and APC-labeled ACSA2 (130-117-386, Miltenyi Biotec). The cells were then rinsed in PBS, centrifuged, resuspended in FACS buffer (1% FBS + 2 mM EDTA, 25 mM HEPES, 1:500 RNA inhibitor in PBS), and incubated in 7AAD (420403, BioLegend) for 10 min before sorting. Data were analyzed using the BD FACS Diva v8.0.1 software. Sorted cells were centrifuged at 400×*g* for 10 min and pellets were lysed in RLT-buffer (74004, Qiagen) for RNA extraction.

### RNA extraction, quantification, and qualification

RNA was isolated from flow-cytometry-sorted cell populations by using an RNeasy Micro Kit (74004, Qiagen) according to the manufacturer’s instructions, which included a step involving incubation with DNase. For whole-brain RNA purification, we generated 1 brain/pool samples. Purified RNA was quantified using a NanoDrop 2000 (Thermo Scientific) and Agilent Technologies Bioanalyzer 2100 RNA Pico chips (5067-1513, Agilent Technologies), according to manufacturer instructions; the RNA integrity number (RIN) in all cases was > 9.

### Preparation of smart-seq2 RNA-seq libraries and sequencing

For RNA sample preparations, 10 ng of RNA per sample was used as the input material. Libraries were generated using a SMART-Seq v4 Ultra Low Input RNA Kit (634892, Takara Bio USA, Mountain View, CA, USA), following the manufacturer’s recommendations, and index codes were added to attribute sequences to each sample. Briefly, first-strand cDNA synthesis from total RNA was primed using 3′ SMART-Seq CDS Primer II A, and SMART-Seq v4 Oligonucleotide was used for template switching at the 5′ end of the transcript. PCR Primer II A was used to amplify cDNA, for 8 cycles, from the SMART sequences introduced by 3′ SMART-Seq CDS Primer II A and the SMART-Seq v4 Oligonucleotide. LD-PCR-amplified cDNA was purified through immobilization on AMPure XP beads and then quantified using the Agilent Bioanalyzer 2100 system. To prepare cDNA libraries suitable for Illumina sequencing, ~ 200 pg of the cDNA was used with a Nextera XT DNA Library Preparation Kit (Illumina, Cat. Nos. FC-131-1024 and FC-131-1096, San Diego, CA, USA). Tagmented fragments were amplified for 12 cycles and dual indexes were added to each well to uniquely label each library. Concentrations were assessed using a KAPA Library Quantification Kit (KK4844, KAPA, Biosystems, USA), and samples were diluted to ~ 2 nm and pooled. Pooled libraries were sequenced on an Illumina NovaSeq platform and 150-bp paired-end reads were generated.

### RNAscope and image quantification

Mice were deeply anesthetized using pentobarbital, transcardially perfused with ice-cold PBS until the irrigation fluid was completely clear, and then perfused with ice-cold 4% paraformaldehyde (PFA) for 10 min. Brains were removed, fixed in 4% PFA in 4 °C refrigerator for 12 h, dehydrated using an ethanol dilution series, embedded in molds containing Tissue-Tek OCT, and frozen in dry ice. The OCT-embedded brain samples were cut into 16-μm coronary sections that were placed onto Fisherbrand Superfrost Plus microscope slides (Thermo Fisher Scientific; 12-550-15). RNAscope experiments were performed using a Manual Fluorescent Multiplex kit v2 (323100, ACDbio), following the manufacturer’s recommendations. Briefly, slices were incubated with hydrogen peroxide and then target retrieval was performed in a boiling bath beaker. Next, protease digestion was performed for 20 min at room temperature by using Protease III for fixed frozen tissues, provided in the kit, after which probe hybridization was conducted for 2 h at 40 °C. A dual-probe set containing Mm-Itgam-c3 (311491) and Mm-Slc1a3-c3 (430781) served as the common probe in each set, and the companion probes were Mm-Cxcl10-c1 (408921) and Mm-Ptbp1-c1 (588721). Nuclei were visualized using 4′,6-Diamidino-2-phenylindole (DAPI).

For each mouse, 3 images per region (technical replicates) were used for the quantification, and 100, 50, and 50 cells were counted in the cortex, hippocampus, and cerebellum, respectively. Images were captured as Z-stacks by using a 20 × objective (NA 0.8) and then maximum-intensity projections were obtained. Lipofuscin autofluorescence was imaged in the blank channel (488 nm) and subtracted from the red channel (594 nm) and far-red channel (647 nm) images. Microglia and astrocytes were identified as RNAscope puncta generated from the Itgam and Slc1a3 probes. Lastly, a blind counting was performed to analyze the number of double-positive cells and the target-probe dots per cell, with each data point representing the mean ± SD of 3 brain slices for each probe set. Then H-score was calculated as follows: H-score $={\sum}_{0-4}^{\mathrm{score}}\left(\mathrm{score}\times \mathrm{percentage}\ \mathrm{of}\ \mathrm{microglia}\ \mathrm{or}\ \mathrm{astrocytes}\right)$. The weighting formula used for the scores is shown in Additional file 1. All parameters were maintained constant between images to allow unbiased detection.

### Quantitative RT-PCR validation of selected genes

Flow-cytometry-sorted microglia and astrocytes were used for RNA extraction (see preceding sections on FACS and RNA extraction). Quantitative RT-PCR was performed in triplicate in 96-well plates by using a qPCR machine (LC480, Roche) and SYBR Green I Master mixture (4887352001, Roche) for detection of amplification products. The following thermocycling protocol was used: initial denaturation at 95 °C for 10 min, followed by 40 amplification cycles of 95 °C for 15 s and 60 °C for 1 min, and a final cycle at 25 °C for 15 s. Relative quantification of mRNA expression was performed using the comparative cycle method to obtain the following ratio: gene of interest/*Gapdh*. Relative quantification of gene-expression levels was performed using the 2^-ΔΔCt^ method. All primers were designed using NCBI Primer-BLAST; we designed primers to be ~ 200-bp long. All primers are listed in Additional file 2.

### STEM (Short Time-series Expression Miner) analyses

The Short Time-series Expression Miner (STEM) is a Java program for clustering, comparing, and visualizing short time series gene expression data from microarray experiments (~ 8 time points or fewer). STEM allows researchers to identify significant temporal expression profiles and the genes associated with these profiles and to compare the behavior of these genes across multiple conditions. The output gene expression value is normalized according to the first time point, usually by subtracting the gene expression value at the first time point, allowing different genes to be visualized at the same starting point. STEM is available for download for free to academic and non-profit users at http://www.cs.cmu.edu/~jernst/stem.

### Graphs and statistical analyses

All statistical analyses were performed using the GraphPad Prism 8.00 software (GraphPad Software, La Jolla, CA, USA). Most data were analyzed using one-way ANOVA followed by Dunnett post hoc test for comparisons of > 3 samples, and two-sample unpaired *t* tests were used for comparing 2 samples; *p* < 0.05 was considered statistically significant.

### Sequencing data quantification and data analysis

#### Quality control

Raw data (raw reads) in fastq format were first processed using in-house perl scripts. In this step, clean data (clean reads) were obtained by removing reads containing adapter sequences, poly-N-containing reads, and low-quality reads from the raw data. Concurrently, Q20, Q30, and GC content of the clean data were calculated. All the downstream analyses were based on the high-quality clean data.

#### Read mapping to reference genome

Reference genome and gene-model annotation files were downloaded from the genome website directly. An index of the reference genome was built and paired-end clean reads were aligned to the reference genome by using Hisat2 v2.0.5. We selected Hisat2 as the mapping tool because Hisat2 can generate a database of splice junctions based on the gene-model annotation file, and thus can yield superior mapping results as compared to other non-splice mapping tools.

#### Quantification of gene-expression level

Feature Counts v1.5.0-p3 was used to determine the number of reads mapped to each gene, after which each gene’s FPKM (the expected number of fragments per kilobase of transcript sequence per million base pairs sequenced) was calculated based on the length of the gene and the number of reads mapped to the gene. FPKM calculation concurrently considers the effect of sequencing depth and the gene length for the read counts, and is currently the most commonly used method for estimating gene-expression levels.

#### Differential expression analysis

Differential expression analysis involving 50 conditions/groups (3 biological replicates per condition) was performed using DESeq2 R package (1.16.1). DESeq2 provides statistical routines for determining differential expression in digital gene-expression data by using a model based on negative binomial distribution. The resulting *p* values were adjusted using the Benjamini and Hochberg approach to control for the false-discovery rate. Genes identified using DESeq2 that featured an adjusted *p* value of < 0.05 were regarded as differentially expressed genes (DEGs).

#### Gene ontology (GO) and Kyoto Encyclopedia of Genes and Genomes (KEGG) enrichment analyses of DEGs

GO enrichment analysis of DEGs was implemented using clusterProfiler R package, in which gene-length bias was corrected; GO terms featuring a corrected *p* value of < 0.05 were considered significantly enriched. KEGG is a database resource for understanding high-level functions and utilities of biological systems, such as of the cell, organism, or ecosystem, from molecular-level information, particularly large-scale molecular datasets generated using genome sequencing and other high-throughput experimental technologies (http://www.genome.jp/kegg/). We used clusterProfiler R package to test the statistical enrichment of DEGs in KEGG pathways.

## Results

### Purification of microglia and astrocytes and RNA-seq profiling

**Fig. 1:**
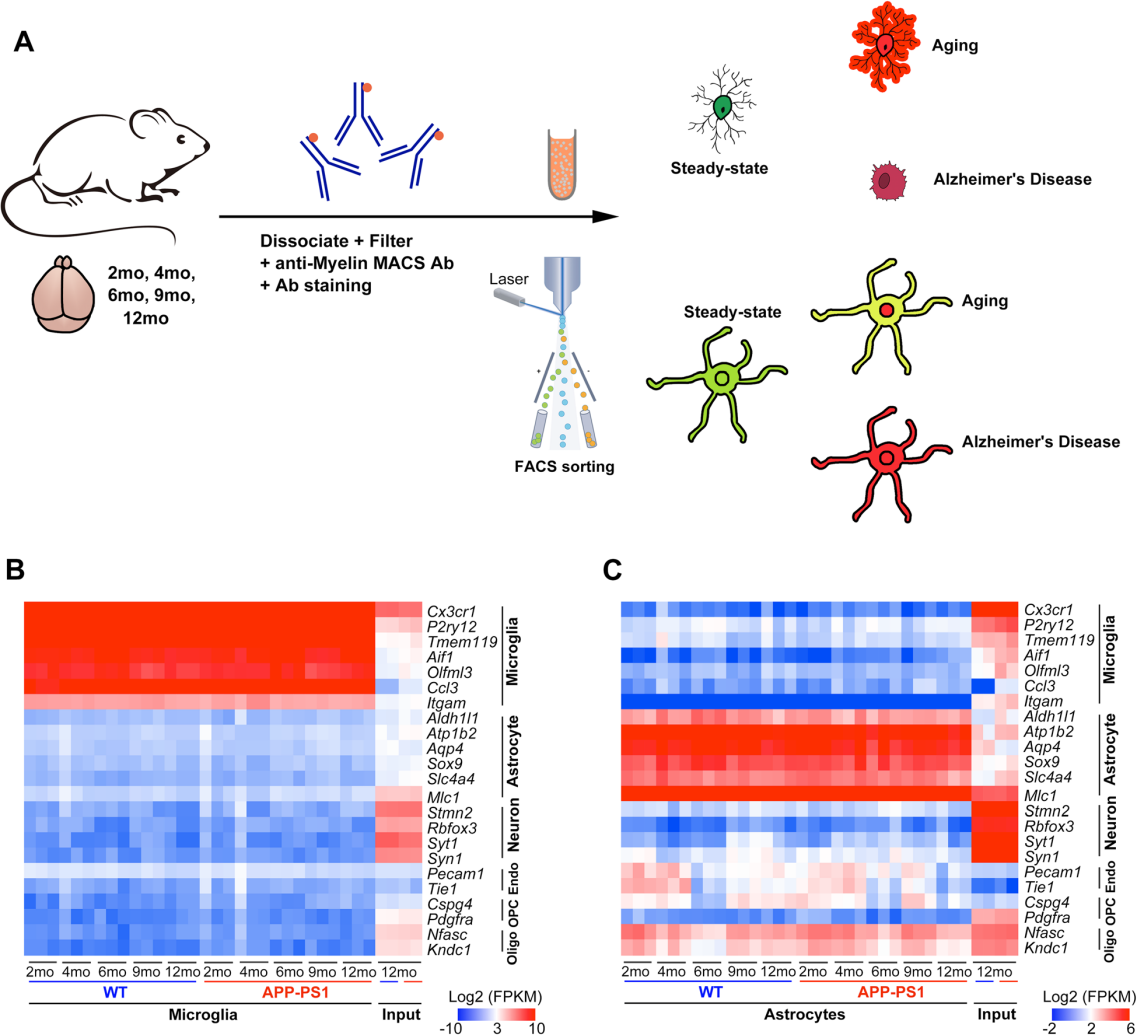
Isolation and purification of microglia and astrocytes. **a** Schematic showing experimental strategy for isolation and sorting of microglia and astrocytes. **b** and **c** RNA-seq analysis of expression of classic cell-specific markers of microglia, astrocytes, neurons, endothelial cells (Endo), oligodendrocyte precursor cells (OPC), and oligodendrocytes (Oligo) in purified microglia (**b**) and astrocytes (**c**), as compared with input (total mRNA). Three biological replicates of each time point (microglia and astrocytes) and 2 biological replicates of input are shown

To investigate whether microglial and astrocyte genes in mice are altered throughout aging, we performed RNA-seq at 5 time points encompassing the mature-adult stage (2 months), when developmental changes in gene expression have ceased, and the middle-age stage (4 months, 6 months, 9 months, 12 months), during which age-dependent pathology develops (Fig. 1a). Microglia were sorted based on CD45^Low-to-Intermediate^/CD11b expression and astrocytes were sorted based on ACSA2 expression after exclusion of doublets and Live/Dead analysis by using BDAriaIII. Single staining and isotype-control antibodies were included as controls (Additional file 3 A-B). The percentage of microglia and astrocytes at different time points was shown in Additional file 4. No significant difference was found for the percentage of the microglia and astrocytes overtime, indicating that that aging or AD has less effect on the cell composition.

We used RNA-seq to assess mRNA purity and quantity. For each time point, microglia and astrocytes were isolated from whole-brain samples, with 3 replicates being included. Furthermore, we sequenced WT/AD whole-brain input samples (2 mice each) to verify the enrichment of microglia and astrocytes, and we developed a transcriptome list of well-known cell-type-specific genes for microglia (e.g., *Cx3cr1*, *P2ry12*, *Tmem119*, *Aif1*, *Olfml3*, *Ccl3*, *Itgam*), astrocytes (e.g., *Aldh1l1*, *Atp1b2*, *Aqp4*, *Sox9*, *Slc4a4*, *Mlc1*), neurons (e.g., *Stmn2*, *Rbfox3*, *Syt1*, *Syn1*), endothelial cells (Endo) (e.g., *Pecam1*, *Tie1*), oligodendrocyte precursor cells (OPC) (e.g., *Pdgfra*, *Cspg4*), and oligodendrocytes (Oligo) (e.g., *Nfasc*, *Kndc1*). Markers specific for microglia were expressed at high levels only in microglia and were undetectable or expressed at extremely low levels in the remaining cell types (Fig. 1b). Despite the high degree of enrichment of astrocyte-specific genes in the majority of samples, we found a small increase in certain neuronal, endothelial, and oligodendrocyte gene contaminants in our astrocyte RNA-seq samples (Fig. 1c). As reported previously, oligodendrocyte markers were detectable as low-level contaminants in adult ACSA2+ astrocytes. These results highlight the importance of validation.

Next, we performed DESeq2 analysis on microglia and astrocytes, which revealed that both cell types showed a gradual increase in the number of age-associated DEGs (40 and 59 genes in 2-month AD microglia and astrocytes as compared to 2-month WT) (Additional file 5A-B). Therefore, we used 2-month WT mice as our mature-adult control for the follow-up analysis, in which DESeq2 R package was used to analysis polyA-selected mRNAs from microglia and astrocytes isolated from whole-brain samples, and we mapped > 85% of the reads in the case of all samples. The reproducibility between replicates was high (Additional file 6A), and the results of principal component analysis (PCA) showed a clear separation of expression between the different time points (Additional file 6B).

### Microglia genes changed upon aging include cytokine-pathway genes

**Fig. 2:**
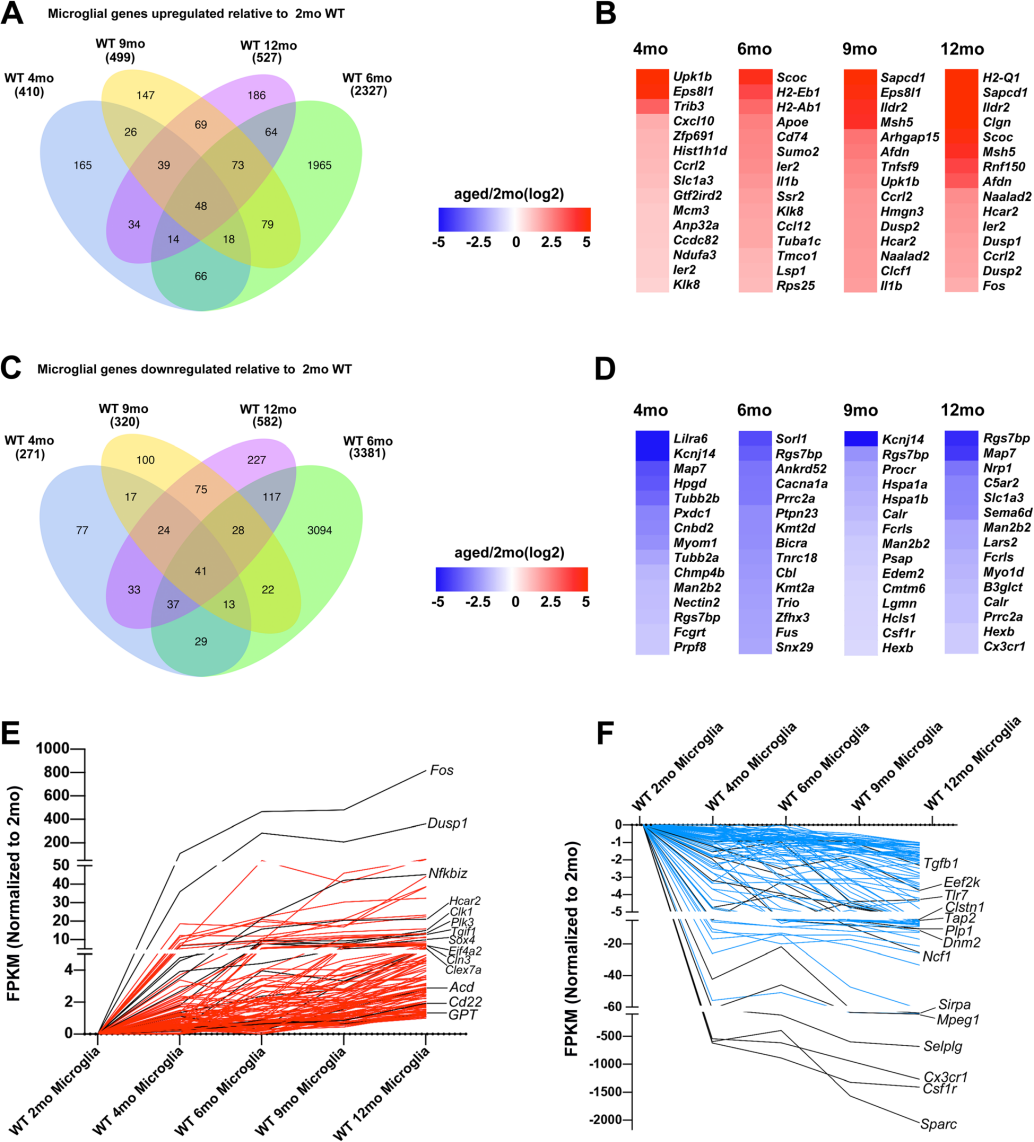
Differential gene expression between adult and aging microglia. **a–d** Upregulated and downregulated genes, determined using DESeq2 analysis, between mature-adult mice (2 months) and aging mice (4 months, 6 months, 9 months, 12 months); adjusted *p* < 0.05, |log_2_ fold-change| > 0.5. **a** Venn diagram showing upregulated genes in microglia. **b** Heatmap of top 15 genes upregulated in microglia. **c** Venn diagram showing downregulated genes in microglia. **d** Heatmap of top 15 genes downregulated in microglia. **e** and **f** STEM analysis of upregulated genes (**e**) and downregulated genes (**f**) in microglia during aging

**Fig. 3:**
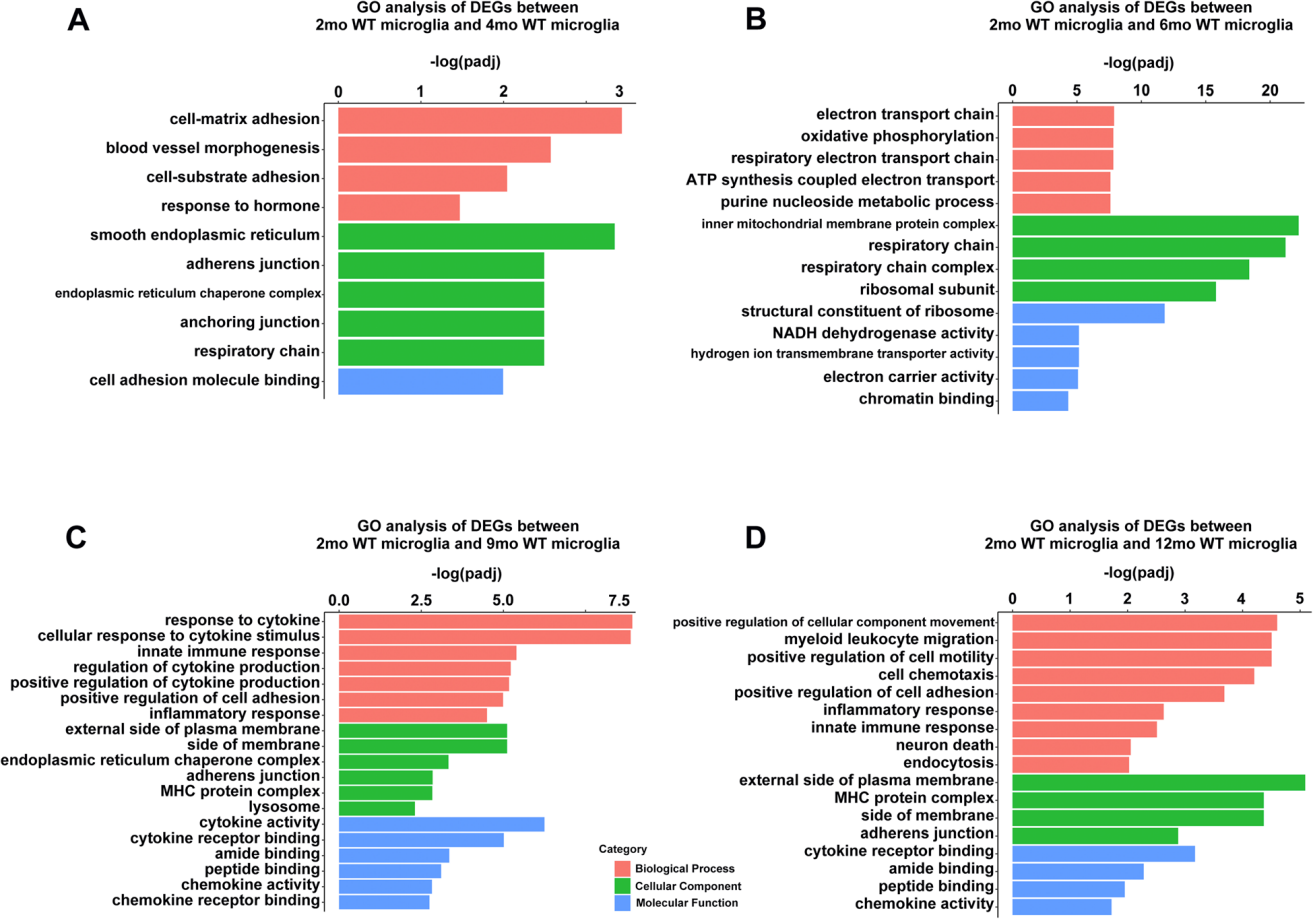
GO analysis of age-related genes between (4 months, 6 months, 9 months, 12 months) WT and (2 months) WT in microglia samples. **a**, **b**, **c**, **d** Representative GO results of age-related genes between 4- (**a**), 6- (**b**), 9- (**c**), 12- (**d**), and 2-month WT in microglia samples

We first determined the number of DEGs (adjusted *p* < 0.05, |log_2_ fold-change| > 0.5) in the aging groups relative to 2-month control. We identified numerous DEGs in aging WT mice in comparison with 2-month WT controls: 1109 genes in 12-month mice, 819 genes in 9-month mice, 5709 genes in 6-month mice, and 681 genes in 4-month mice (Fig. 2). Compare to 2-month WT controls, the top 15 upregulated genes exclusively in 4, 6, 9, and 12 months microglia are shown in Fig. 2 b. To annotate these genes in different biological pathways, we performed GO and KEGG analysis. As compared with the expression in 2-month microglia, we detected altered genes involved in “blood vessel morphogenesis” and “cell-matrix adhesion” in 4-month microglia (Fig. 3a); “oxidative phosphorylation” and “ATP metabolic process” in 6-month microglia (Fig. 3b); “response to cytokine” and “innate immune response” in 9-month microglia (Fig. 3c); and “positive regulation of cellular component movement” and “chemotaxis” in 12-month microglia (Fig. 3d). Additional file 7 shows the complete datasets.

In this study, we hypothesized that age-dependent pathogenic or protective genes could be expressed at consistently higher or lower levels throughout the different time points of mice as compared with the expression in the 2-month control. Therefore, we constructed a Venn diagram of the genes consistently upregulated in aging microglia (4 months, 6 months, 9 months, and 12 months, relative to 2-month control), and from this we identified 48 genes (termed “age-up” microglial genes) (Additional file 8 shows the gene list with fold change and *p* values), which included a cassette of genes involved in the cytokine pathway. *Cxcl10* was upregulated 3-, 1.9-, 3.5-, and 3-fold in 4-month, 6-month, 9-month, and 12-month microglia relative to the expression in 2-month microglia. This result was further confirmed using RNAscope in situ hybridization. Other age-up microglial genes involved in immunoregulatory and inflammatory processes included *Ccl2/Ccl12*, *Egr2*, *Nr1d2*, *Il6*, *Zfp36* (anti-inflammatory signaling), *Nfkbia* (negative regulation of NFεB transcription factor activity), *H2-Q1* (MHC I protein-complex member), and *Ccrl12*.

We next applied the aforementioned filtering criteria to identify genes that are downregulated in microglia. Fewer genes were downregulated than those upregulated in microglia, and considerably more genes were differentially expressed in 6-month microglia (3381 genes) than those in 4-month, 9-month, and 12-month microglia (271, 320, and 582 genes, respectively) (Fig. 2c). Compare to 2-month WT controls, the top 15 downregulated genes exclusively in 4-, 6-, 9-, and 12-month microglia are shown in Fig. 2d. We identified 41 “age-down” microglial genes (Additional file 9 shows the gene list with fold change and *p* values), including well-known genes such as *Man2b2*, which encodes lysosomal acid α-D-mannosidase [24]; *CYFIP1*, which encodes a protein that functions in cytoskeletal remodeling to ensure proper dendritic-spine formation [25, 26]; *Wasf2*, another cytoskeleton regulator [27]; the inflammation-driven cancer gene *Ptbp1* [28]; and toll-like receptor genes (*Tlr5*, *Tlr9*). We also used STEM (Short Time-series Expression Miner) analysis [29] to cluster gene sets that showed dynamic changes over time (Fig. 2e, f and Additional file 10). The gene alterations included the upregulation of *Fos* [30] and *cd22* [31] and the downregulation of *Csf1r* (colony-stimulating factor 1 receptor gene) [32] and *Cx3cr1* (C-X3-C motif chemokine receptor 1 gene) [33–35]. Previous studies showed that microglia age-related genes do not differ among brain regions [36], so the significant changed microglia genes might not be brain region specific.

### Astrocyte genes showing age-dependent upregulation include synaptic-transmission-regulating genes and peptidase-inhibitor genes

**Fig. 4:**
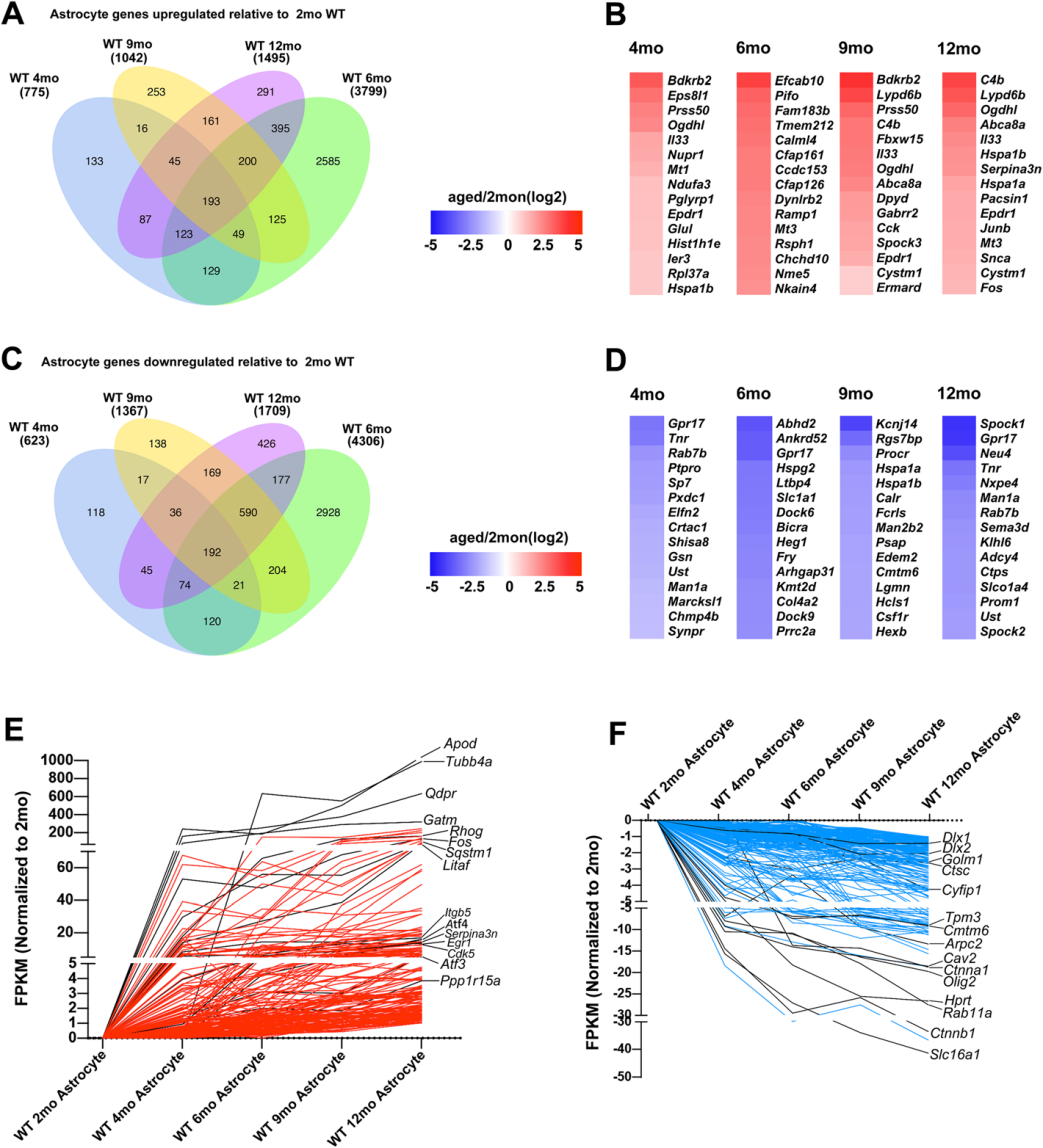
Differential gene expression between adult and aging astrocytes. **a**–**d** Upregulated and downregulated genes, determined using DESeq2 analysis, between mature-adult mice (2 months) and aging mice (4 months, 6 months, 9 months, 12 monts); adjusted *p* < 0.05, |log_2_ fold-change| > 0.5. **a** Venn diagram showing upregulated genes in astrocytes. **b** Heatmap of top 15 genes upregulated in astrocytes. **c** Venn diagram showing downregulated genes in astrocytes. **d** Heatmap of top 15 genes downregulated in astrocytes. **e** and **f** STEM analysis of upregulated genes (**e**) and downregulated genes (**f**) in astrocytes during aging

**Fig. 5:**
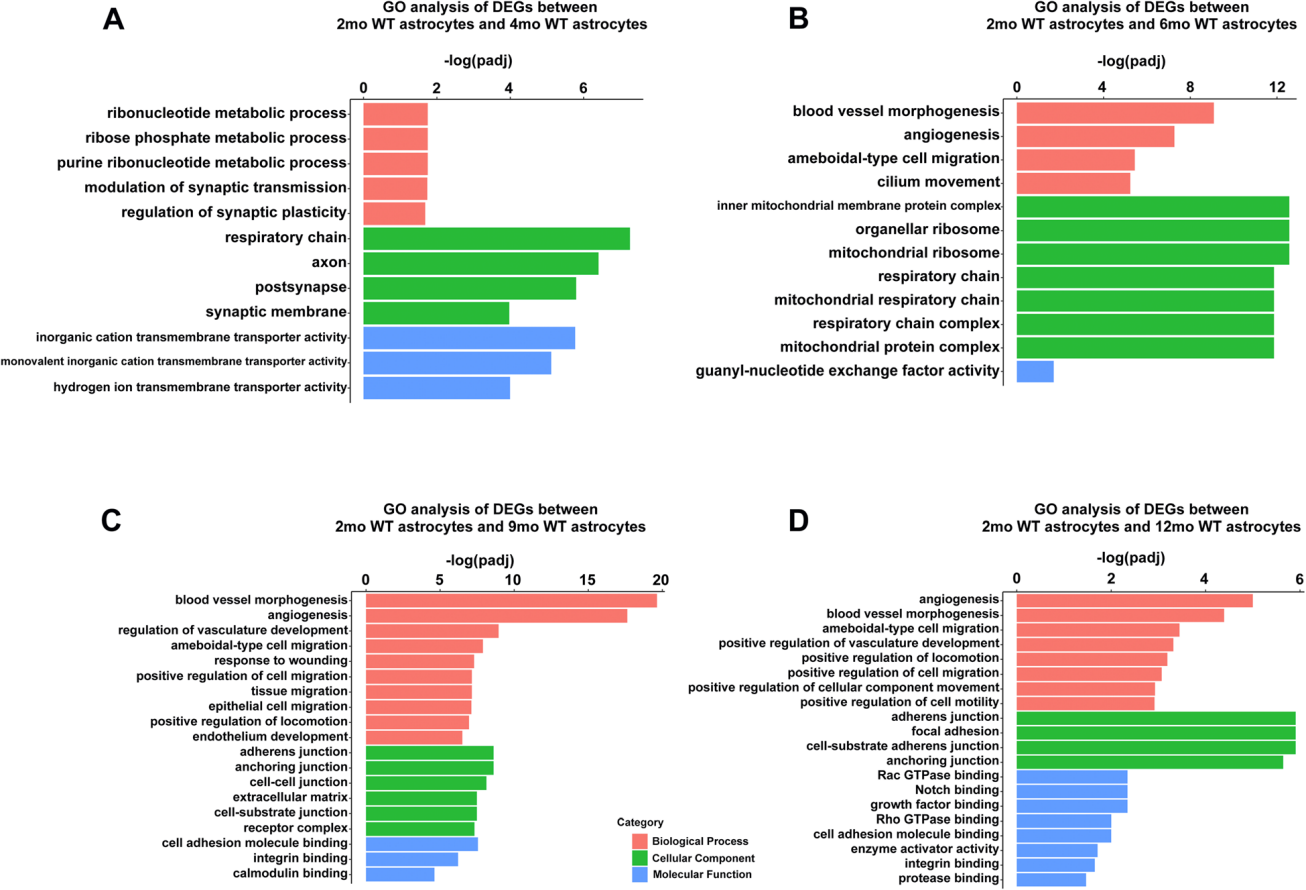
GO analysis of age-related genes between (4 months, 6 months, 9 months, 12 months) WT and (2 months) WT in astrocytes samples. **a**, **b**, **c, d** Representative GO results of age-related genes between 4- (**a**), 6- (**b**), 9- (**c**), 12- (**d**), and 2-month WT in astrocytes samples

We next compared gene expression between astrocytes from young mice (2 months) and aging mice (4 months, 6 months, 9 months, 12 months), which revealed age-dependent variations in the expression levels (Fig. 4a, c). Compare to 2-month WT controls, the top 15 upregulated and downregulate genes exclusively in 4-, 6-, 9-, and 12-month astrocytes are shown in Fig. 4b,and d. As compared with the expression in 2-month astrocytes, the representative GO results showed that DEGs involved in “ribonucleotide metabolic process” in 4-month astrocytes (Fig. 5a); “blood vessel morphogenesis” and “blood vessel morphogenesis” in 6-month astrocytes (Fig. 5b); “epithelial cell migration” in 9-month astrocytes (Fig. 5c ); and “adherens junction” and “enzyme activator activity” in 12-month astrocytes (Fig. 5d) (Additional file 11 shows the complete GO and KEGG datasets).

The reactive-astrocyte markers Serpina3n and Osmr were expressed at higher levels in 12-month astrocytes than in 2-month astrocytes, whereas *Il33* expression showed significant differences in all comparisons. Specifically, 193 genes were upregulated (“age-up” astrocyte genes) (Additional file 12 shows the gene list with fold change and *p* values), including a well-known AD risk gene (*Apoe*) and a gene encoding a component of the complement cascade (*C4b*). Moreover, *Snca* (synuclein-α) and *Sncg* (synuclein-γ) were also upregulated throughout aging.

We also found that age-up genes included several peptidase-inhibitor genes. Two of these genes, *Spock3* and *Timp4*, were upregulated 1.5–3.8-fold throughout aging. Cst3, an endogenous cysteine-protease inhibitor [37–40], was upregulated in the early stages of aging; the expression was increased 2.2-, 3.7-, 1.6-, and 1.9-fold in 4-month, 6-month, 9-month, and 12-month astrocytes relative to that in 2-month astrocytes. Among the age-up astrocyte genes, we also noted significant upregulation of the gene encoding Pcsk1n. We identified 192 genes that are downregulated in astrocytes (“age-down” astrocyte genes) were also identified in this study (Additional file 13), which included some of the genes involved in negative regulation of axon extension, such as Tnr, Nrp1, Ptprs, Slit1, and Sema4f. In addition, it also included a matrix metallopeptidase (Mmp16). The STEM analysis results are shown in Fig. 4e and f and Additional file 10.

To estimate whether the changed genes in astrocytes are brain region specific, we compared our age-altered astrocytes gene dataset to the genes which are uniquely up/downregulated in astrocytes in different brain regions published before [22]. We found that astrocyte genes shifted their regional expression patterns upon aging. The age-up genes were upregulated in different brain regions, including 37 genes in cerebellum, 6 in visual cortex, 16 in hypothalamus, and 2 in all brain regions (Additional file 14 A-B). Interestingly, age-down genes were also downregulated in brain regions, such as 69 in cerebellum, 13 in motor cortex, 16 in visual cortex, and 102 in hypothalamus (Additional file 14 C-D). Importantly, we also found that the human [36] and mouse astrocyte genes affected by aging shared 17 orthologous genes (Additional file 14 E). Cross-sectional genes were shown in Additional file 15.

Most DEGs were altered exclusively in either microglia or astrocytes. However, 4 genes were included among both age-up microglial genes and age-up astrocyte genes: *Cxcl10*, *Ccl2*, *Scoc*, which regulate amino acid-starvation-induced autophagy [41], and *Mri1*, which is involved in the methionine salvage pathway. Conversely, 7 genes were downregulated in both microglia and astrocytes: *Man2b2*, *Ptbp1*, *Prrc2a*, which control oligodendroglial specification and myelination by functioning as a newly identified m^6^A reader [42]; *Midn*, which regulates glucokinase enzyme activity [43]; *Fscn1*, which is required for filopodial formation in neural crest cells [44]; *Clcn6*, which is related to voltage-gated chloride channel activity; and *Pik3r4*, which is involved in the formation of autophagosomes [45].

### Interaction of microglia and astrocytes during aging and AD

Previous study by Liddelow et al. [46] showed that activated microglia induced A1 astrocytes by secreting Il-1α, TNF, and C1q, also happening in normal aging [47]. In the progression of WT and AD, we also found that inflammatory inducer cytokines secreted by microglia appeared earlier than the upregulation of neuroinflammatory genes in A1-like reactive astrocytes (Additional file 16), indicating that microglia might induced A1 astrocytes in aging and AD progression.

### Validation of RNA-seq profiles by using qPCR and RNAscope

**Fig. 6:**
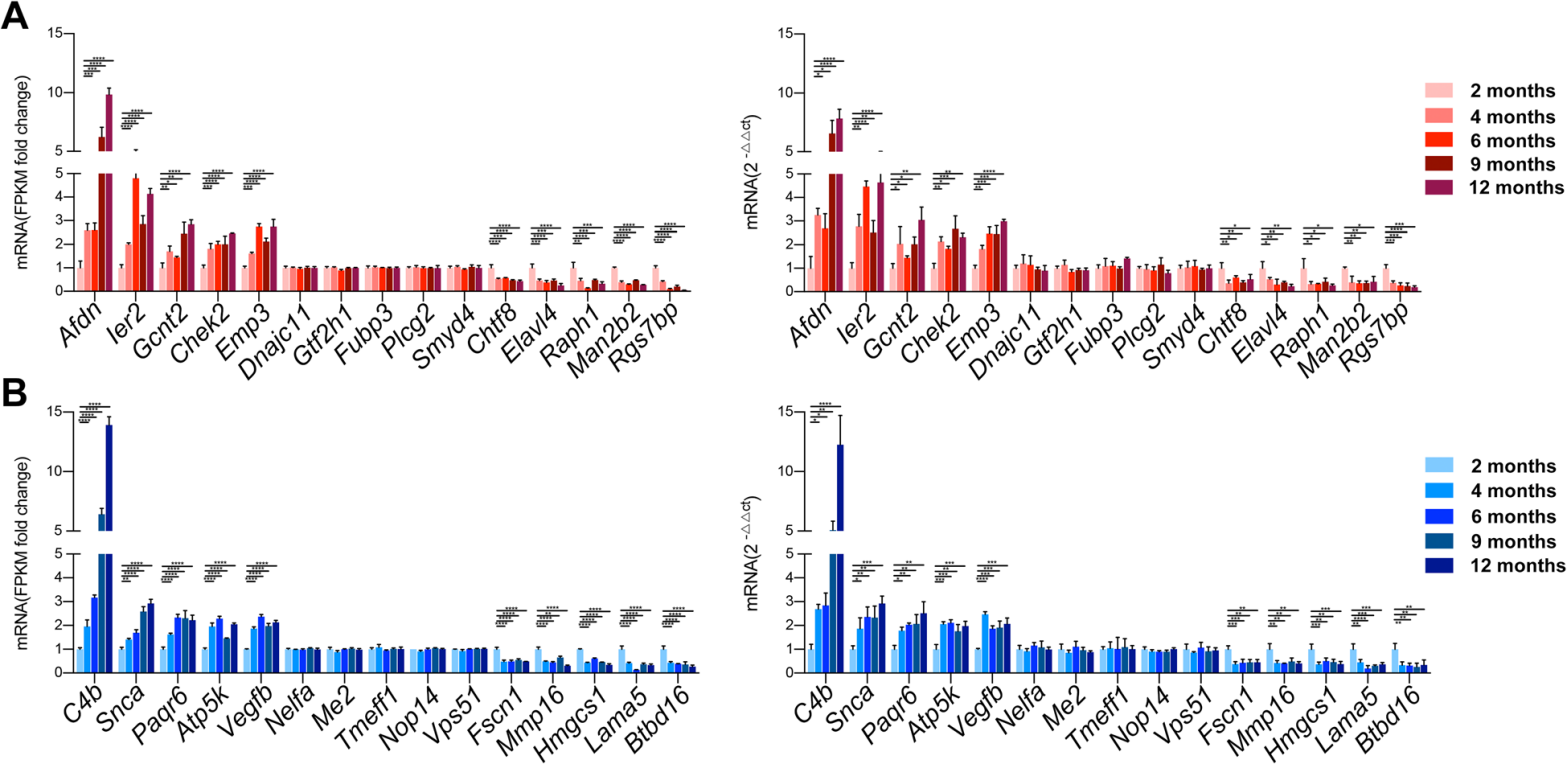
Validation of RNA-seq data between 5 time points WT samples. **a** Expression analyses performed on selected genes yielded results superimposable with results obtained from RNA-seq analyses of microglia. **b** Expression analyses performed on selected genes yielded results superimposable with results obtained from RNA-seq analyses of astrocytes. Columns represent means ± SEM; ****p < 0.0001, ***p < 0.001, **p < 0.01, *p < 0.05; left: comparisons of DESeq2-analysis values between 2-month and aging samples (4 months, 6 months, 9 months, 12 months); right: unpaired *t* tests for comparing 2 samples

We validated our RNA-seq data through qPCR performed using a new cohort of animals. For age-altered genes, we selected 15 genes from microglia and astrocytes respectively (5 age-up genes, 5 genes expressed no difference in age, 5 age-down genes). For each time point of WT/AD mice, we selected 9 genes from microglia and astrocytes (3 showing elevated expression in 2-month WT mice, 3 equally expressed, and 3 showing increased expression in 2-month AD mice; genes for 4 months, 6 months, 9 months, and 12 months were selected in a similar manner). Data were expressed as 2^-ΔΔCt^ by using the *Gapdh* transcript as an internal reference standard. The expression analyses performed on the selected genes yielded results that were superimposable with the results obtained using RNA-seq (Fig. 6 and Additional file 17).

**Fig. 7:**
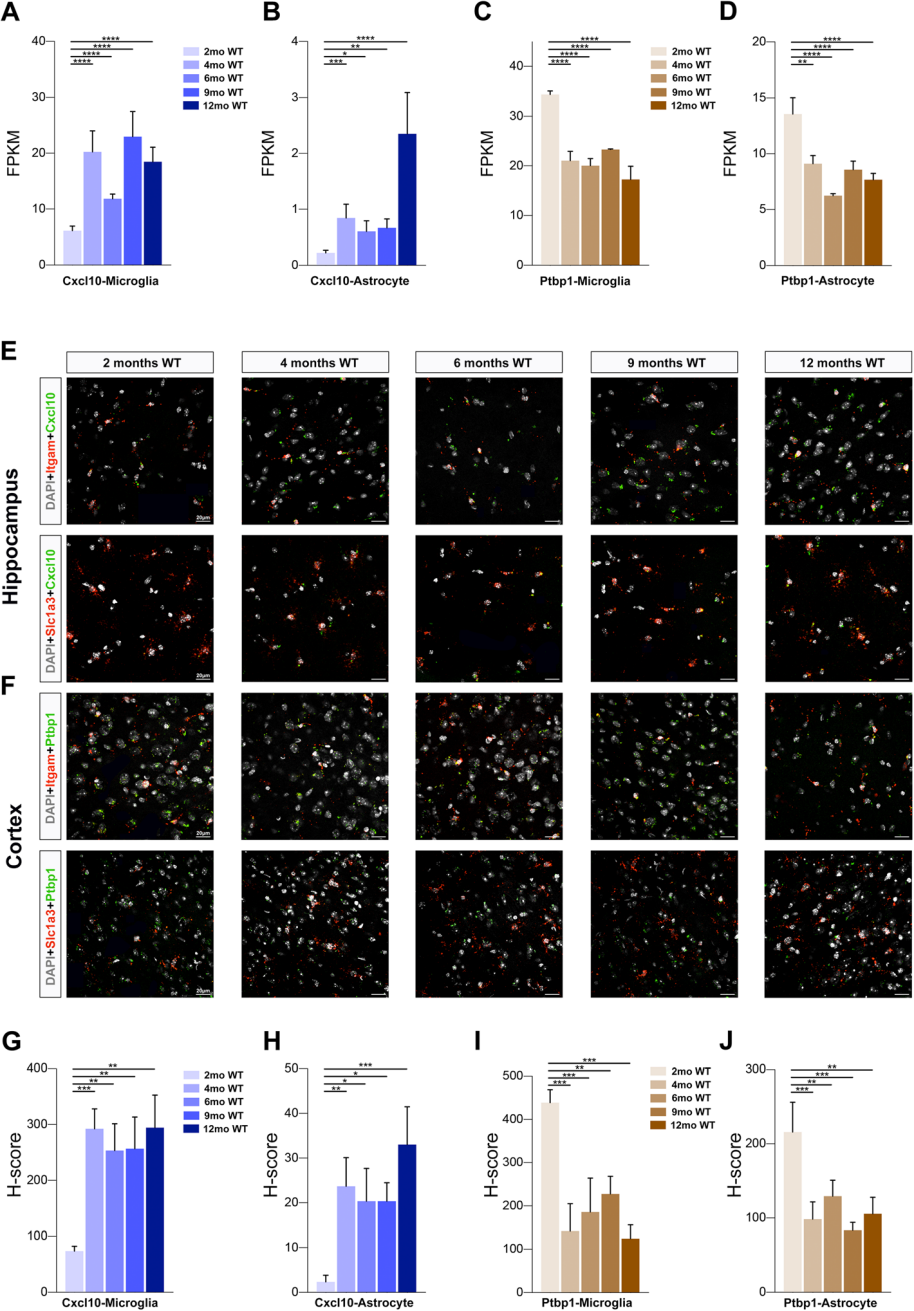
Validation of aging-induced changes in microglial and astrocyte gene expression by using in situ hybridization. **a**–**d** Bar graphs of RNA-seq data showing FPKM values determined for Cxcl10 in microglia (**a**), Cxcl10 in astrocytes (**b**), Ptbp1 in microglia (**c**), and Ptbp1 in astrocytes (**d**) upon aging. Columns represent means ± SEM; ****p < 0.0001, ***p < 0.001, **p < 0.01, *p < 0.05, for comparisons of DESeq2-analysis values between 2-month and aging samples (4 months, 6 months, 9 months, 12 months). **e** and **f** Representative in situ hybridization images for Cxcl10 (**e**) and Ptbp1 (**f**) showing colocalization with a microglial marker (Itgam) and astrocyte marker (Slc1a3) in cortex and hippocampus in 2-month, 4-month, 6-month, 9-month, and 12-month mice. Scale bar, 20 μm. **g**–**j** Bar graphs depicting quantification of H-score of Itgam + microglia and Slc1a3 + astrocytes expressing detectable levels of Cxcl10 and Ptbp1 mRNAs upon aging: (g) Cxcl10 in microglia, (**h**) Cxcl10 in astrocytes (**i**), Ptbp1 in microglia, and (**j**) Ptbp1 in astrocytes. One-way ANOVA followed by Dunnett post hoc test; data are shown as means ± SEM; ****p < 0.0001, ***p < 0.001, **p < 0.01, *p < 0.05; *n* = 3 animals

To confirm mRNA changes in the case of age-up genes from microglia and astrocytes, we performed dual RNAscope in situ hybridization on samples from WT mice belonging to the 5 age groups. We used Itgam (CD11b) as a universal microglial marker and Slc1a3 as a universal astrocyte marker, and we examined an age-up gene (*Cxcl10*) and an age-down gene (*Ptbp1*) from the RNA-seq analysis (Fig. 7e, f). We determined the total dual-positive cell numbers for Itgam^+^ microglia or Slc1a3^+^ astrocytes that also expressed Cxcl10 or Ptbp1 in the hippocampus, cortex, and cerebellum, as well as the number of target-probe dots per cell. We found a similar fold-change in FPKM as in the RNA-seq data (Fig. 7a–d, g–f): *Cxcl10* and *Ptbp1* were significantly upregulated and downregulated, respectively, with age in both microglia and astrocytes.

### Association of age-altered genes in AD transcriptomes

**Fig. 8:**
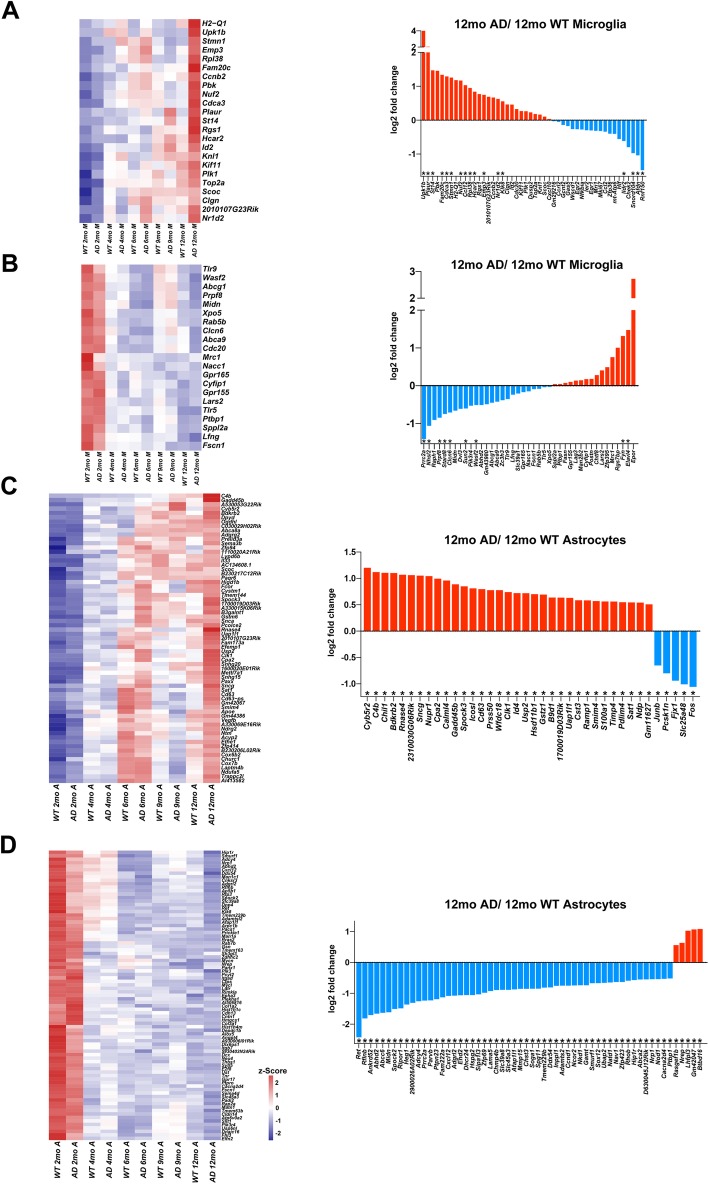
Age-related gene-expression and variation between AD (12 months) and WT (12 months) mice. **a**–**d** (left), Heatmap represent the age-up microglial gene (**a**); age-down microglial gene (**b**); age-up astrocyte gene (**c**), and age-down astrocyte gene at 5 time points WT/AD samples. *Z* scores are calculated from gene FPKM values (upregulation in red, downregulation in blue, neutral in white). **a**–**d** (right), Age-up microglial gene (**a**); age-down microglial gene (**b**); age-up astrocyte gene (**c**), and age-down astrocyte gene (**d**) expression between AD (12 months) and WT (12 months) samples. Log_2_ fold-change based on RNA-seq data, between 12-month AD and 12-mo WT mice; *adjusted *p* < 0.05, |log_2_ fold-change| > 0.5. **c** and **d** (right) Only genes that exhibited statistically significant changes in expression are shown

AD is a heterogeneous disease in which multiple detrimental factors contribute to cognitive loss and disease escalation [14]. To determine the aging transcriptomes in AD, we analyzed the expression of age-altered genes at 5 different time points in WT and AD mice. We found that most age-up genes were highly expressed in AD mice as compared to WT mice at the same time points. In contrast, age-down genes were highly expressed in WT mice (Fig. 8). Then we compared the expression of age-altered genes in microglia or astrocytes isolated from 12-month WT vs. 12-month AD mice. We found that among the 28 age-up genes in microglia from 12-month AD mice, 13 showed a significant increase (adjusted *p* < 0.05, |log_2_ fold-change| > 0.5) relative to the age-matched control (Fig. 8a), whereas 4 of the 28 genes were downregulated. Among the age-down microglial genes, 7 genes were significantly downregulated in AD mice (Fig. 8b). We also analyzed age-related astrocyte genes in AD progression, and we found that 33 age-up genes were strongly upregulated and 53 age-down genes were markedly downregulated in 12-month AD mice (Fig. 8c, d). As shown in Additional file 18, we identified several cross-changed genes between the DEGs (genes are altered in both aging and Alzheimer’s disease) and the DEGs (genes are altered in different AD groups compared with 2-month AD controls).

### Nonmonotonically changed age-related genes

**Fig. 9:**
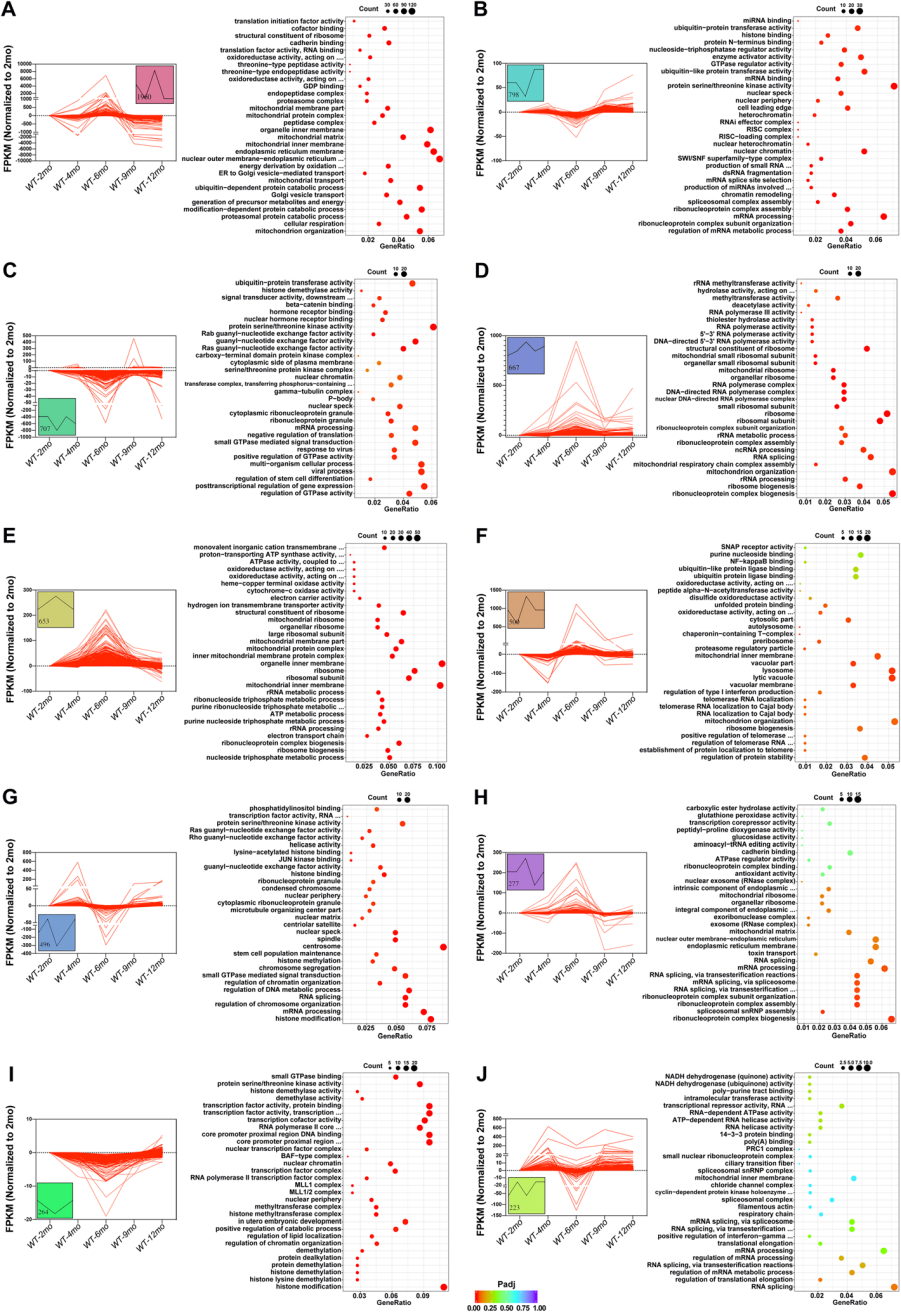
STEM and GO analysis of age-related microglial DEGs clustered by the same pattern. **a**–**g** (left), STEM analysis of age-related microglial DEGs clustered by the same pattern. Trends in genes with the same pattern are shown in the box and the number represents the number of genes. **a**–**g** (right), GO analysis of the genes shown in left

We used STEM analysis to cluster gene sets that showed similar trends in a certain category at 5 time points between 2 and 12 months. As shown in Fig. 9a, the expression of genes was upregulated sharply from 4 months, peaked at 6 months, downregulated again, and stabilized at 9 months. The GO analysis showed that DEGs mainly involved in “mitochondrion organization” and “cellular respiration”. The DEGs were downregulated from 2 to 6 months and then upregulated are shown in Fig. 9i. These differentially expressed genes were involved in histone modification pathway. Other trends in microglial genes with the same pattern are shown in Fig. 9.

**Fig. 10:**
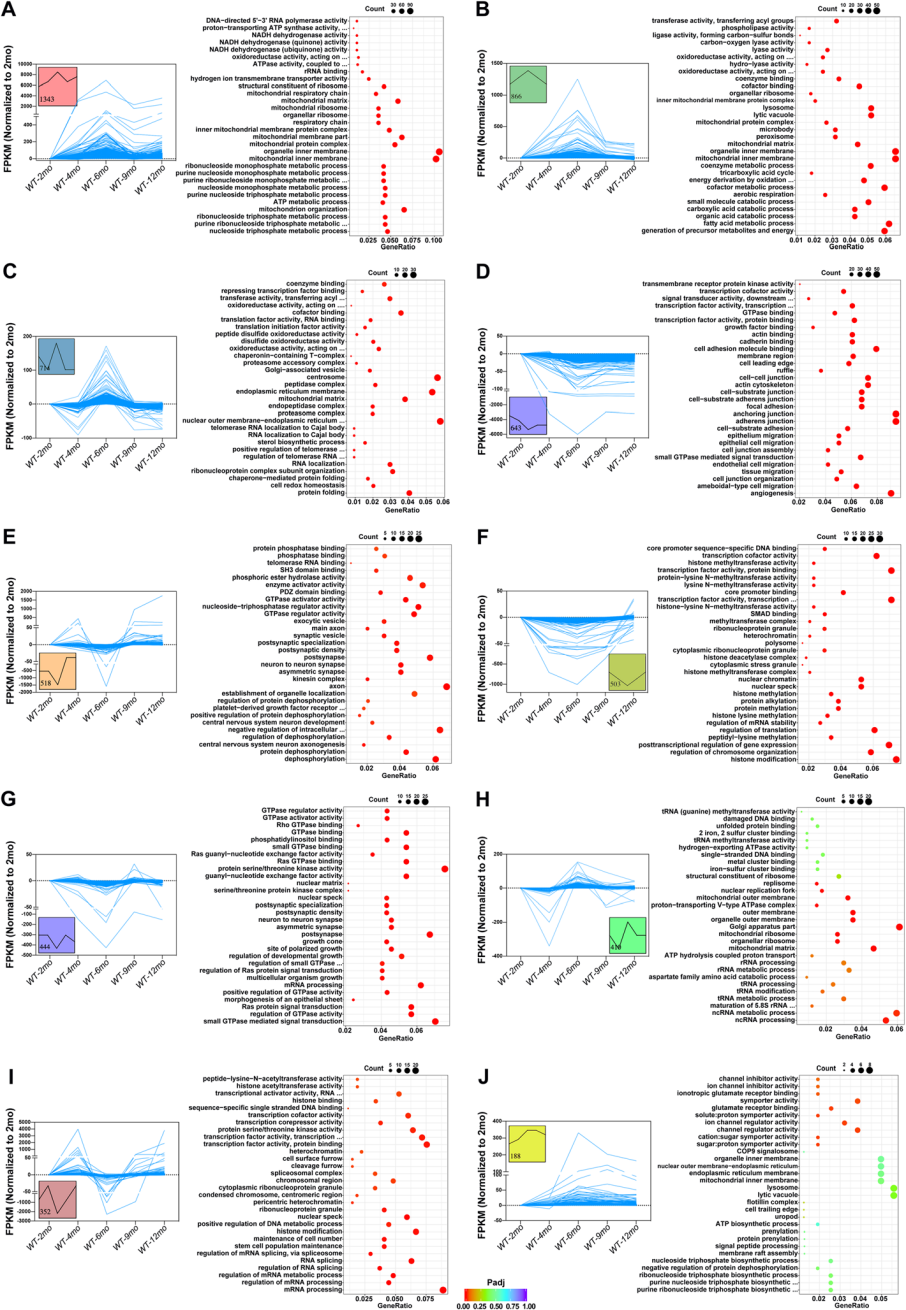
STEM and GO analysis of age-related astrocyte DEGs clustered by the same pattern. **a**–**g** (left), STEM analysis of age-related astrocyte DEGs clustered by the same pattern. Trends in genes with the same pattern are shown in the box and the number represents the number of genes. **a**–**g** (right), GO analysis of the genes shown in left

Similarly, we also found that a cassette of genes in astrocytes at different time points showing a same variation tendency. We performed GO analysis on these fixed trend genes and the results were shown in Fig. 10. “Transcription cofactor activity” (Fig. 10d, e), “mitochondrial protein complex/membrane/matrix” (Fig. 10a, b, c, and h) and other pathways were involved in different patterns of DEGs, indicating that they may play different roles at different stages.

## Discussion

The results of this study indicate that microglia exhibit an increase in responsiveness to inflammation stimuli with age, which is reflected by the consistently elevated expression of inflammatory-response genes, whereas astrocytes appear to function as “preservers” of inflammation, which is reflected by the upregulation of peptidase-inhibitor genes upon aging. In this study, we tried our best to reduce the artificial effect of the dissociation process. Although there are three biological replicates at each time point, individual differences still cannot be ignored. In this study, we have not found a significant difference of the percentage of microglia and astrocytes overtime.

Transcriptome differences between microglia and astrocytes during aging have been addressed in a few previous studies. Aged astrocytes (2 years old) were shown to upregulate genes involved in synapse elimination but to minimally alter the expression of homeostasis-related genes [22], and 2-year-old astrocytes were also reported to adopt the reactive phenotype of neuroinflammatory A1-like reactive astrocytes [47]. Our astrocyte gene-expression dataset agrees with the findings of the previous studies, because we also detected a significant decrease in *Thbs1*, an increase in *Thbs2*, *C4b*, *Cxcl10*, and reactive-astrocyte genes (*Osmr*, *Serpina3n*), and no change in homeostasis genes (*Aldh1l1*, *Aqp4*) in our 12-month astrocyte samples relative to the expression in 2-month astrocytes. Similarly, the DEGs identified between our 12- and 2-month microglial samples broadly agreed with previous RNA-seq profiles of microglia from the aged brain (24 months). Age-up genes in astrocytes, *C4b,* involved in synapse elimination [48]; *Snca* and *Sncg*, 2 genes related to Parkinson’s disease pathogenesis [49] that also play several roles in synaptic activity, such as regulation of synaptic-vesicle trafficking and subsequent neurotransmitter release [50, 51], suggesting that astrocytes play a critical role in synapse elimination and synaptic transmission. *Spock3* and *Timp4*, encode proteins that participate in inhibiting matrix metalloproteinases (MMPs) involved in the degradation of the extracellular matrix [52], and the other genes encode proteins that inhibit lipoteichoic acid-induced NF-κB, MAP kinase, and Akt activities [53] and decrease the invasion and metastasis of tumor cells in the brain [54]. Pcsk1n, an inhibitor of prohormone convertase 1 that regulates the proteolytic cleavage of neuroendocrine peptide precursors [55]. Cst3, can function as a protective factor in the AD brain, and its mechanisms of action include inhibition of cysteine proteases, induction of autophagy, induction of cell division, and inhibition of Aβ oligomerization and amyloid-fibril formation [56–59]. Cst3 is known to be enriched in adult astrocytes throughout the brain [60], but the role of Cst3 in astrocytes during aging has remained obscure. Inhibition of proteolysis has been shown to protect neurons against ischemia [61, 62], which suggests that astrocytes might protect neurons by commonly upregulating the expression of the aforementioned glial-cell-derived endogenous protease inhibitor. Previous studies [63] have reported that when the blood-brain barrier is destroyed, peripheral leukocytes are able to transit the astrocytic TJ barrier in inflammatory lesions and enter the CNS, secrete serine proteases and MMPs to cleave astrocytic CLDN4. Our results suggest that in normal aging, increased production of serine proteases inhibiter/MMPs inhibiter and decreased production of MMPs might control neuroinflammation and prevent the invasion of peripheral pathogens.

Although we minimize ex vivo activation during the isolation procedure, there may still be some unknown activation when sorting cells from the brain. Evidence has been presented supporting the notion that the inflammatory responses of both astrocytes and microglia peak during the beginning of symptomatology [64]. Therefore, we focused here on the genes that start to change at an early stage and show sustained alteration throughout aging. Unexpectedly, we found sustained upregulated or downregulated expression in microglia throughout life of several inflammation-related genes. Ccl2 is a key mediator of spinal microglial activation, and blocking spinal Ccl2 alleviates heat hyperalgesia and augments glutamatergic transmission in substantia gelatinosa neurons [65], reduces immunosuppression, and augments vaccine immunotherapy [66]. Ccl12 also plays a pivotal role during the early stages of allergic lung inflammation [67, 68]. These findings suggest that some of the predisposing or inflammatory factors associated with diseases might be related to the consistently elevated expression of Ccl2/Ccl12 in microglia, and blocking this might alleviate and minimize disease progression. Egr2, which has been proposed as a newly identified M2 (alternatively activated) marker for macrophages, is associated with the ability of these cells to respond to inflammatory stimuli [69, 70], was included among the age-up microglial genes. Intriguingly, we found an increase in Nr1d2 (also known as REV-ERBβ), which acts as a nodal output of the circadian clock and thus links cellular circadian timers with innate-immune responses and thereby modulates the production and release of the proinflammatory cytokines Ccl2 and IL-6 [71–74]. *Nr1d2* was increased 1.6–2-fold and *Il6* was increased 2.2–3-fold among age-up microglial genes, which suggests that Nr1d2 upregulation might stabilize the diurnal variation in Ccl2 and IL-6 levels and immune function caused by aging. Other age-up microglial genes involved in immunoregulatory and inflammatory processes included *Zfp36* (anti-inflammatory signaling), *Nfkbia* (negative regulation of NFεB transcription factor activity), *H2-Q1* (MHC I protein-complex member), and *Ccrl12*. Taken together, these data support an active role for microglia in inflammation response throughout aging. We further found that neuropsin (Klk8), an extracellular matrix serine protease that induces neurite outgrowth and regulates Schaffer-collateral long-term potentiation (LTP) [75, 76], was also significantly increased throughout aging, which suggests that microglia could play a notable role in the establishment of LTP and synaptic plasticity. Over time, altered genes involvement in angiogenesis to subsequent innate immune inflammatory responses, indicates roles of microglia in neuroinflammatory responses during aging.

We compared aging microglia heterogeneity between mice and humans by performing comparisons of our mouse age-related microglial datasets with two previous human microglial aging profile s[77, 78]. Limited overlap was observed in microglial genes regulated during aging between mice and humans, indicating that human and mouse microglia age differently. In addition to doing the analysis relative to the earliest time point (2 months), we also used STEM analysis to cluster gene sets that showed similar trends in a certain pattern. The GO analysis showed that different pathways were involved in different categories such as mitochondrion organization, cellular respiration, or mRNA metabolic process, indicating that different genes and signaling pathways may play certain roles at different stages.

At the early stage (2 months), the number of differentially expressed genes between WT and AD was negligible, and showed a gradual increase with age. When we compared the age-up related genes in WT/AD at the same time points, we found that most age-up genes were highly expressed in AD mice as compared to WT mice. This suggests that age genetic changes in the AD process occur earlier than aging. And the change may also involve in the development of AD. In the later stages of aging, the internal environment of the CNS shows increased complexity, and this might involve the actions of peripheral factors together with BBB dysfunction. Imaging analyses have revealed that the BBB is localized at the level of tight junctions between brain endothelial cells [79]. Aging and AD are both associated with diminished BBB integrity and an opening for T cell transendothelial migration into the CNS [80–82]. In the parenchyma, bidirectional crosstalk occurs between the infiltrating cells and the resident glial cells; activated microglia impair BBB function by releasing several inflammatory modulators and thus lead to hyperpermeability; and the resulting T cell infiltration, in turn, favors increased microglial activation by secreting proinflammatory cytokines or acting in a protective manner toward senescent microglia [3, 17, 20, 83, 84]. Notably, transient early depletion of regulatory T cells was shown to reduce the recruitment of microglia toward amyloid deposits and alter the disease-related gene-expression profile in the brain [85].

## Conclusions

In summary, our study on microglia and astrocytes throughout aging provides new insights into the gene-expression profiles of these 2 types of glial cells and into the age-related changes in the transcriptome in relation to normal aging. Some of the genes identified here to be altered might represent targets for the treatment of the cognitive decline that occurs in diseases associated with aging.

## Supplementary information

**Additional file 1.** The weighting formula of H-score.

**Additional file 2.** Primer list.

**Additional file 3.** Cell isolation by using FACS. A, FACS gating strategy for isolating microglia and astrocytes. B, Gating of isotype control to minimize autofluorescence.

**Additional file 4.** The percentage of microglia and astrocytes at five time points.

**Additional file 5.** Age-dependent increase in number of DEGs. A, Numbers of DEGs determined using DESeq2 analysis between microglia from WT and AD mice (2mo, 4mo, 6mo, 9mo, 12mo). B, Numbers of DEGs determined using DESeq2 analysis between astrocytes from WT and AD mice (2mo, 4mo, 6mo, 9mo, 12mo). Adjusted *p* < 0.05, |log2 fold-change| > 0.5.

**Additional file 6.** Transcriptome profiles of microglia and astrocytes. A, Heatmap of Pearson’s correlation between microglia (left) and astrocytes (right) (2mo, 4mo, 6mo, 9mo, 12mo). B, Principal component analysis (PCA) of RNA-seq samples.

**Additional file 7.** GO and KEGG annotate DEGs between 2mo WT microglia and aging WT microglia (4mo, 6mo, 9mo, and 12mo).

**Additional file 8.** Age-up microglia genes list with fold change and p-values.

**Additional file 9.** Age-down microglia genes list with fold change and *p*-values.

**Additional file 10.** STEM analysis of 5 time points WT microglia and astrocytes and the FPKM expression of genes.

**Additional file 11.** GO and KEGG annotate DEGs between 2mo WT astrocytes and aging WT astrocytes (4mo, 6mo, 9mo, and 12mo).

**Additional file 12.** Age-up astrocyte genes list with fold change and *p*-values.

**Additional file 13.** Age-down astrocyte genes list with fold change and *p*-values.

**Additional file 14.** Comparison of the age-altered astrocyte genes in this study with the region-specific datasets from Matthew et al. [22] and the human datasets from Soreq et al [36]. A, Venn diagram of age-up astrocyte genes and upregulated genes in astrocytes from 4 different brain regions from Matthew et al. B, Venn diagram of age-up astrocyte genes and overlap upregulated genes in astrocytes from 4 all brain regions from Matthew et al. C, Venn diagram of age-down astrocyte genes and downregulated genes in astrocytes from 4 different brain regions from Matthew et al. D, Venn diagram of age-down astrocyte genes and overlap downregulated genes in astrocytes from 4 all brain regions from Matthew et al. E, Venn diagram of age-up astrocyte genes and overlap genes upregulated in human astrocytes from Soreq et al.

**Additional file 15.** Cross-sectional genes in Additional file 14.

**Additional file 16. **Heatmaps comparing the mean expression of *Il1a/TNF* in microglia samples and pan-reactive/A1-specific genes in astrocyte samples. A. Heatmap of the mean expression of *Il1a/TNF* in five time points WT microglia and Cxcl10/Serpina3n in five time points WT astrocytes. B. Heatmap of the mean expression of *Il1a/TNF* in five time points AD microglia and *Cxcl10/Serpina3n/Aspg/Gfap/Fbln5/Fkbp5* in five time points AD astrocytes.

**Additional file 17. **Validation of RNA-seq data between WT and AD samples. A-E, Expression analyses performed on selected genes yielded results superimposable with results obtained from RNA-seq analyses of microglia. F-J, Expression analyses performed on selected genes yielded results superimposable with results obtained from RNA-seq analyses of astrocytes. Columns represent means ± SEM; ****p < 0.0001, ***p < 0.001, **p < 0.01, *p < 0.05; left: comparisons of DESeq2 values between WT and AD samples; right: unpaired *t* tests for comparing 2 samples.

**Additional file 18.** Venn diagram of age-related DEGs in APP/PS1 mice and its relationship with the age-altered DEGs significantly upregulated/downregulated in AD group. A-D, Upregulated/downregulated genes, determined using DESeq2 analysis, between APP/PS1 mice (2mo) and APP/PS1 mice (4mo, 6mo, 9mo, 12mo); adjusted *p* < 0.05, |log2 fold-change| > 0.5. A, Venn diagram showing upregulated genes in microglia (left), core genes and age-altered DEGs significantly upregulated/downregulated in Fig. 8A. B, Venn diagram showing downregulated genes in microglia (left), core genes and age-altered DEGs significantly upregulated/downregulated in Fig. 8B. C, Venn diagram showing upregulated genes in astrocytes (left), core genes and age-altered DEGs significantly upregulated/downregulated in Fig. 8C. D, Venn diagram showing downregulated genes in astrocytes (left), core genes and age-altered DEGs significantly upregulated/downregulated in Fig. 8D.

## Data Availability

Raw and normalized gene-expression data have been deposited in the GEO (GEO: GSE137028).

## Abbreviations

AD: Alzheimer’s disease
WT: Wild-type
CNS: Central nervous system
BBB: Blood-brain barrier
Aβ: Amyloid-β
RNA-seq: RNA-sequencing
PCA: Principal component analysis
DEGs: Differentially expressed genes
GO: Gene ontology
KEGG: Kyoto Encyclopedia of Genes and Genomes
FACS: Fluorescence activated cell sorting
DAPI: 4′,6-Diamidino-2-phenylindole
