# Supplementary figures and images for: Transcriptomic profiling of microglia and astrocytes throughout aging
Source: J Neuroinflammation. 2020 Apr 1;17:97. doi: 10.1186/s12974-020-01774-9 (PMC7115095; doi:10.1186/s12974-020-01774-9)

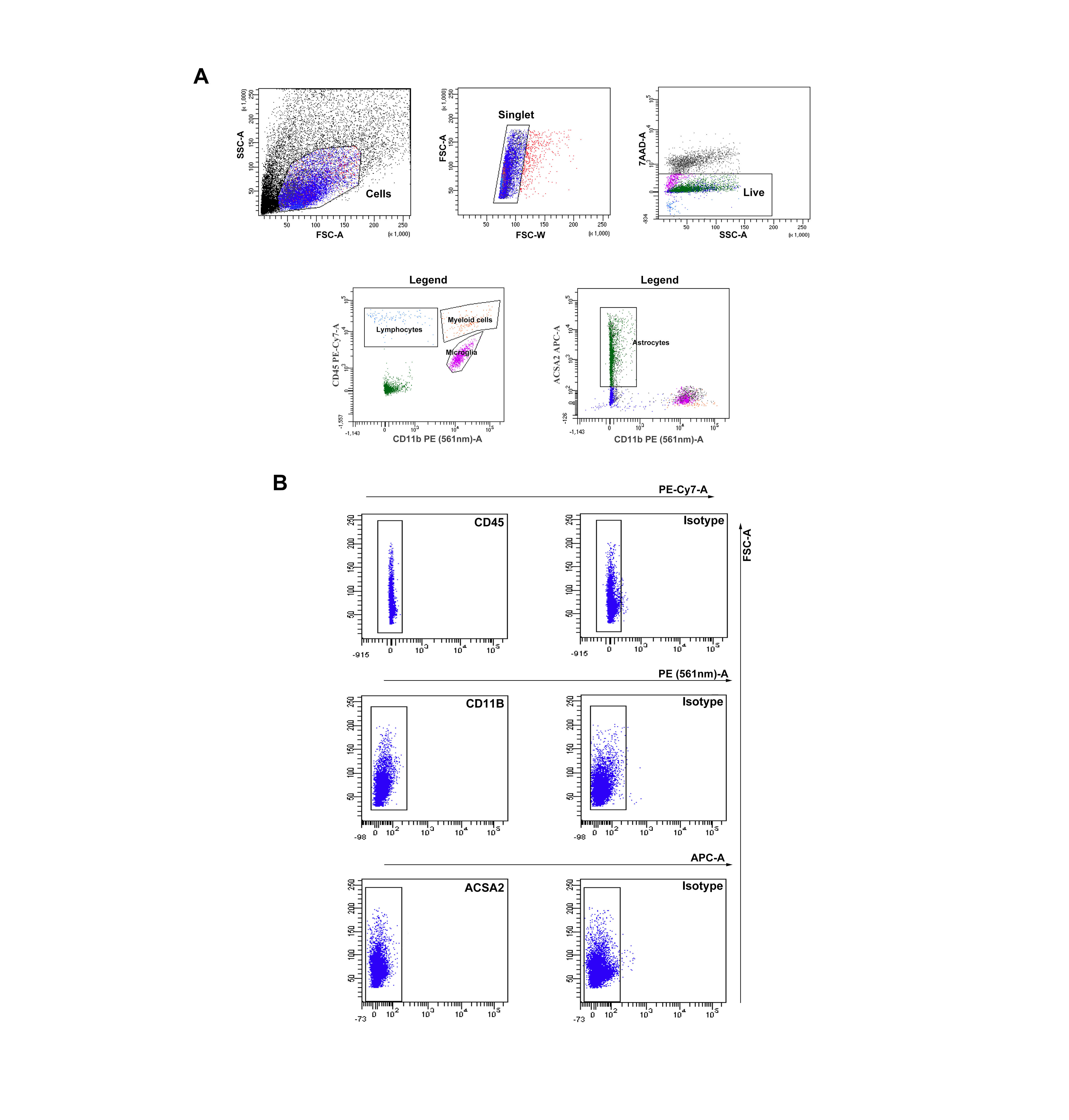

Supplement: Supplementary file 3 — Additional file 3. Cell isolation by using FACS. A, FACS gating strategy for isolating microglia and astrocytes. B, Gating of isotype control to minimize autofluorescence. [file 12974_2020_1774_MOESM3_ESM.tiff]

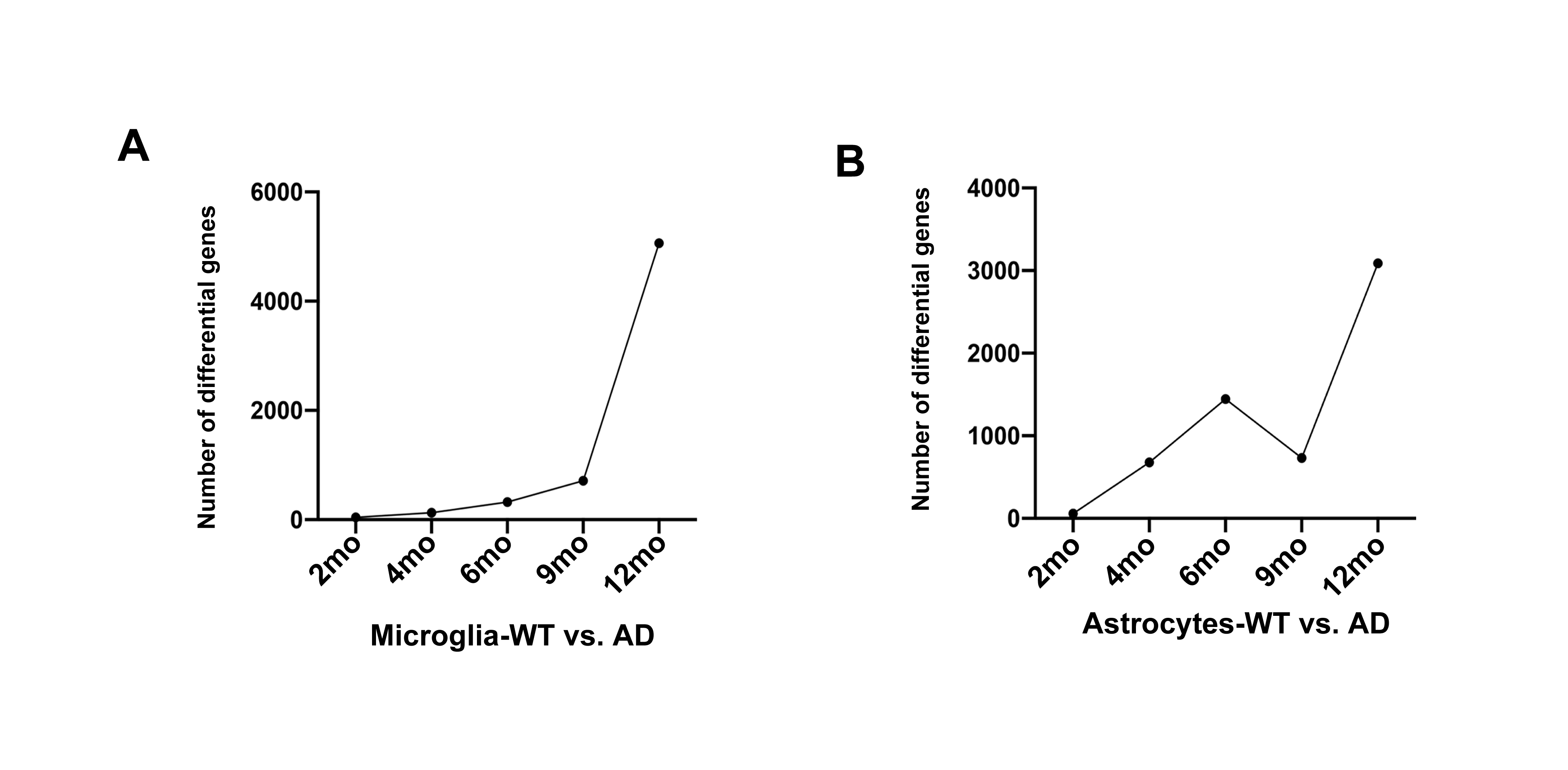

Supplement: Supplementary file 5 — Additional file 5. Age-dependent increase in number of DEGs. A, Numbers of DEGs determined using DESeq2 analysis between microglia from WT and AD mice (2mo, 4mo, 6mo, 9mo, 12mo). B, Numbers of DEGs determined using DESeq2 analysis between astrocytes from WT and AD mice (2mo, 4mo, 6mo, 9mo, 12mo). Adjusted p < 0.05, |log2 fold-change| > 0.5. [file 12974_2020_1774_MOESM5_ESM.tif]

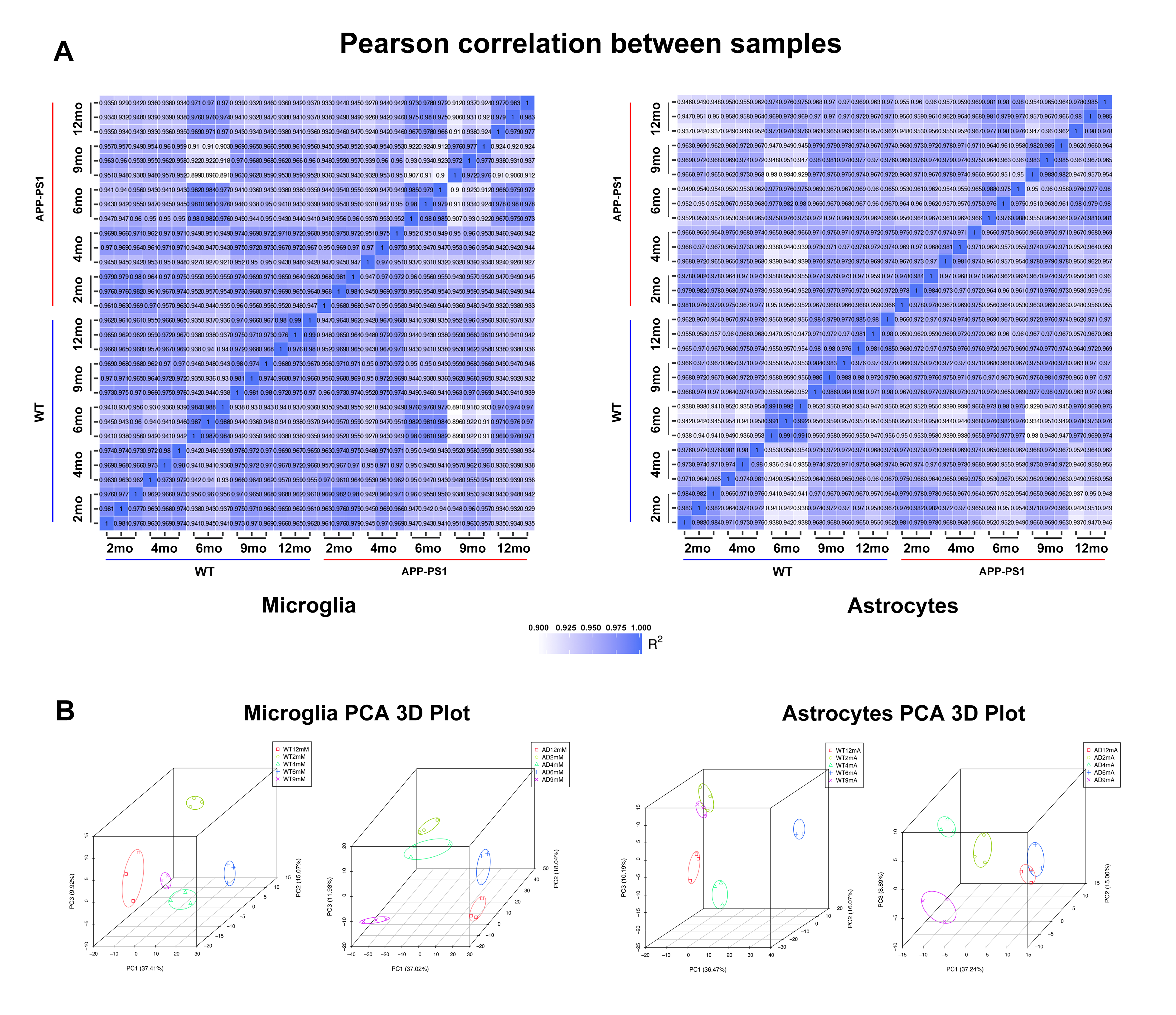

Supplement: Supplementary file 6 — Additional file 6. Transcriptome profiles of microglia and astrocytes. A, Heatmap of Pearson’s correlation between microglia (left) and astrocytes (right) (2mo, 4mo, 6mo, 9mo, 12mo). B, Principal component analysis (PCA) of RNA-seq samples. [file 12974_2020_1774_MOESM6_ESM.tif]

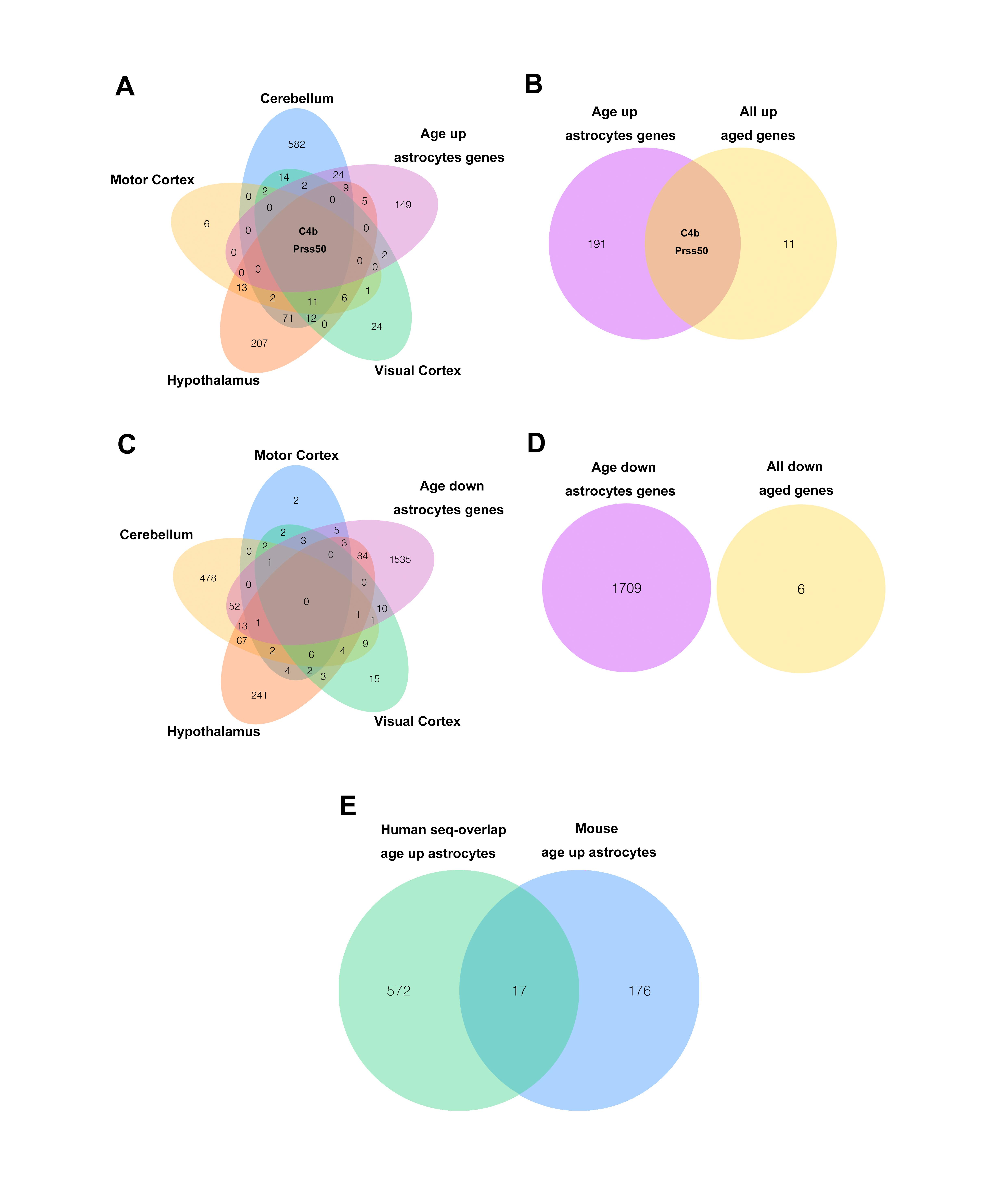

Supplement: Supplementary file 14 — Additional file 14. Comparison of the age-altered astrocyte genes in this study with the region-specific datasets from Matthew et al. [22] and the human datasets from Soreq et al [36]. A, Venn diagram of age-up astrocyte genes and upregulated genes in astrocytes from 4 different brain regions from Matthew et al. B, Venn diagram of age-up astrocyte genes and overlap upregulated genes in astrocytes from 4 all brain regions from Matthew et al. C, Venn diagram of age-down astrocyte genes and downregulated genes in astrocytes from 4 different brain regions from Matthew et al. D, Venn diagram of age-down astrocyte genes and overlap downregulated genes in astrocytes from 4 all brain regions from Matthew et al. E, Venn diagram of age-up astrocyte genes and overlap genes upregulated in human astrocytes from Soreq et al. [file 12974_2020_1774_MOESM14_ESM.tif]

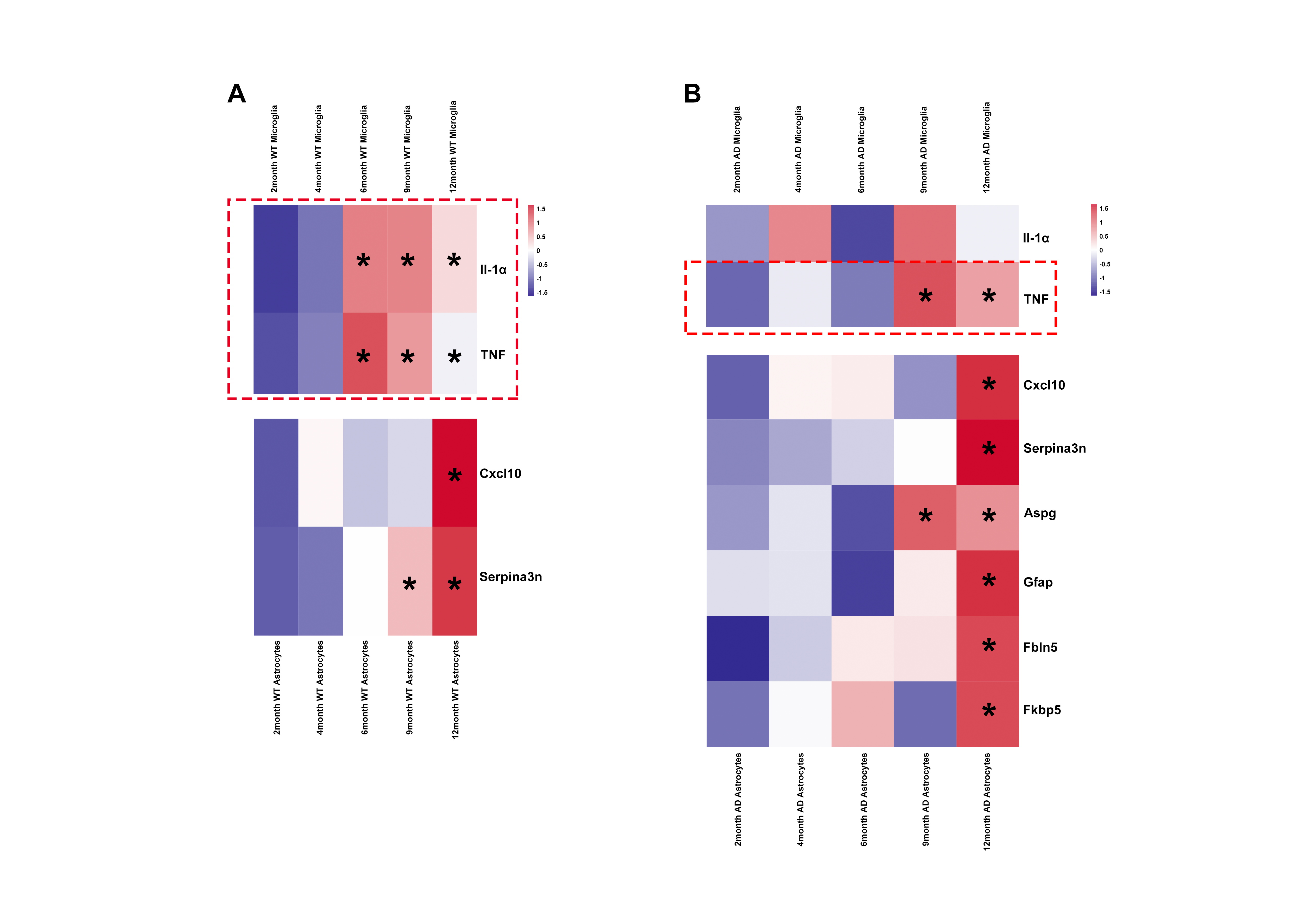

Supplement: Supplementary file 16 — Additional file 16. Heatmaps comparing the mean expression of Il1a/TNF in microglia samples and pan-reactive/A1-specific genes in astrocyte samples. A. Heatmap of the mean expression of Il1a/TNF in five time points WT microglia and Cxcl10/Serpina3n in five time points WT astrocytes. B. Heatmap of the mean expression of Il1a/TNF in five time points AD microglia and Cxcl10/Serpina3n/Aspg/Gfap/Fbln5/Fkbp5 in five time points AD astrocytes. [file 12974_2020_1774_MOESM16_ESM.tif]

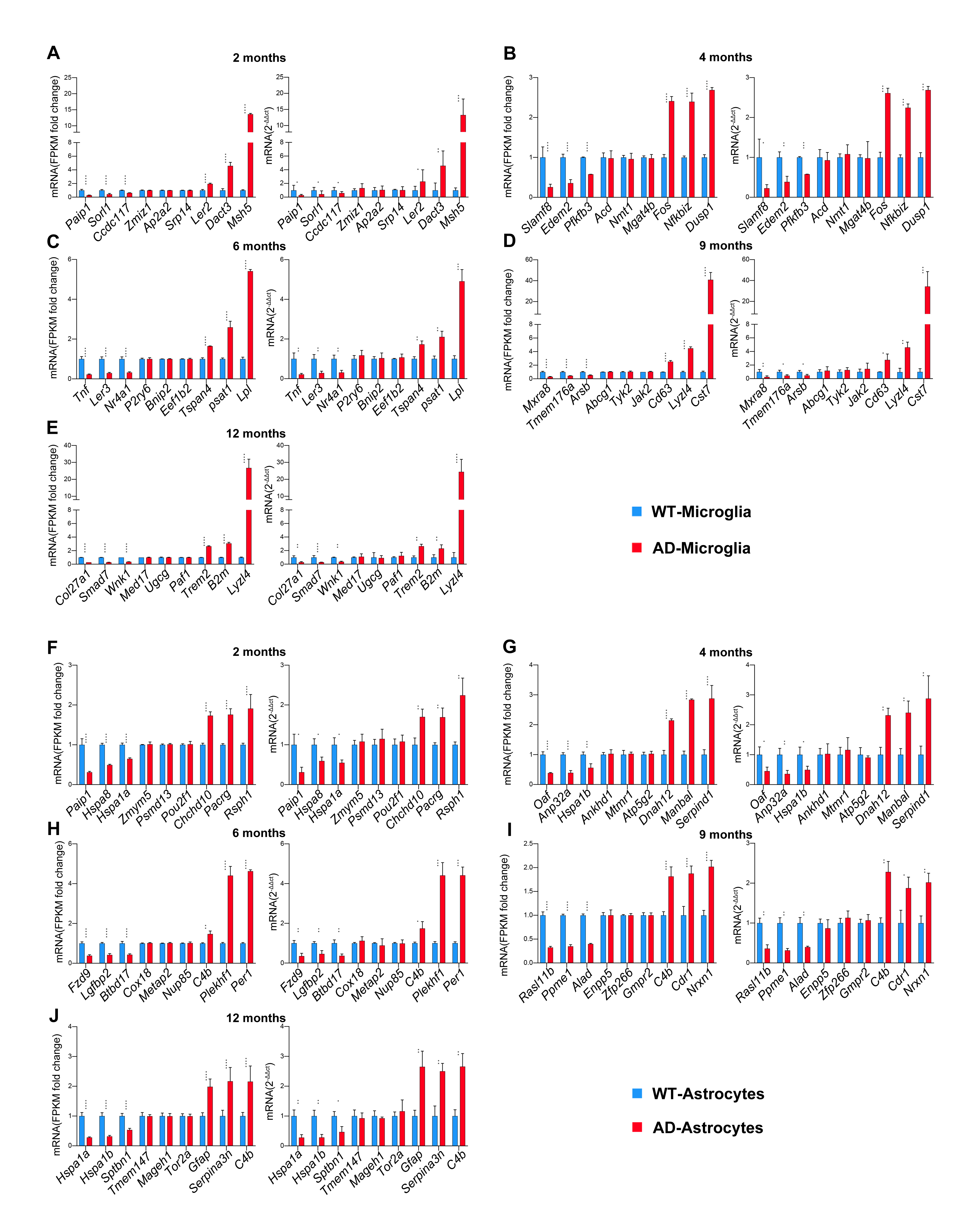

Supplement: Supplementary file 17 — Additional file 17. Validation of RNA-seq data between WT and AD samples. A-E, Expression analyses performed on selected genes yielded results superimposable with results obtained from RNA-seq analyses of microglia. F-J, Expression analyses performed on selected genes yielded results superimposable with results obtained from RNA-seq analyses of astrocytes. Columns represent means ± SEM; ****p < 0.0001, ***p < 0.001, **p < 0.01, *p < 0.05; left: comparisons of DESeq2 values between WT and AD samples; right: unpaired t tests for comparing 2 samples. [file 12974_2020_1774_MOESM17_ESM.tif]

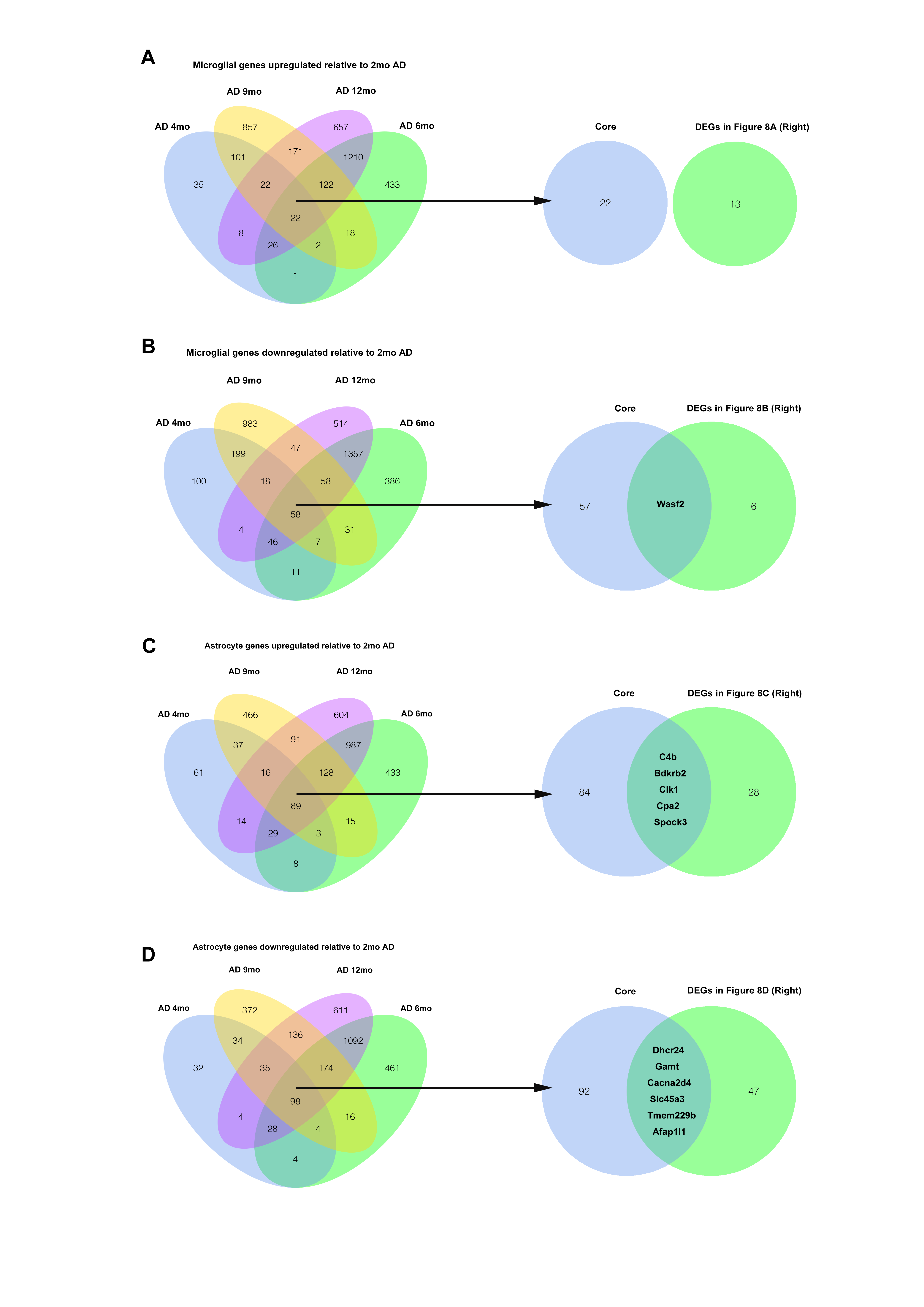

Supplement: Supplementary file 18 — Additional file 18. Venn diagram of age-related DEGs in APP/PS1 mice and its relationship with the age-altered DEGs significantly upregulated/downregulated in AD group. A-D, Upregulated/downregulated genes, determined using DESeq2 analysis, between APP/PS1 mice (2mo) and APP/PS1 mice (4mo, 6mo, 9mo, 12mo); adjusted p < 0.05, |log2 fold-change| > 0.5. A, Venn diagram showing upregulated genes in microglia (left), core genes and age-altered DEGs significantly upregulated/downregulated in Fig. 8A. B, Venn diagram showing downregulated genes in microglia (left), core genes and age-altered DEGs significantly upregulated/downregulated in Fig. 8B. C, Venn diagram showing upregulated genes in astrocytes (left), core genes and age-altered DEGs significantly upregulated/downregulated in Fig. 8C. D, Venn diagram showing downregulated genes in astrocytes (left), core genes and age-altered DEGs significantly upregulated/downregulated in Fig. 8D. [file 12974_2020_1774_MOESM18_ESM.tif]
